# Dynamic metal-ligand coordination for multicolour and water-jet rewritable paper

**DOI:** 10.1038/s41467-017-02452-w

**Published:** 2018-01-09

**Authors:** Yun Ma, Pengfei She, Kenneth Yin Zhang, Huiran Yang, Yanyan Qin, Zihan Xu, Shujuan Liu, Qiang Zhao, Wei Huang

**Affiliations:** 1 Key Laboratory for Organic Electronics and Information Displays & Jiangsu Key Laboratory for Biosensors, Institute of Advanced Materials (IAM), Jiangsu National Synergetic Innovation Center for Advanced Materials (SICAM), Nanjing University of Posts and Telecommunications (NUPT), Nanjing, 210023 China; 2 Key Laboratory of Flexible Electronics (KLOFE) and Institute of Advanced Materials (IAM), Jiangsu National Synergetic Innovation Center for Advanced Materials (SICAM), Nanjing Tech University, Nanjing, 210028 China; 3 Shaanxi Institute of Flexible Electronics (SIFE), Northwestern Polytechnical University (NPU), Xi’an, 710072 China

## Abstract

Rewritable paper has recently become prevalent in both academic research and marketplace due to the potential environmental advantages, including forest conservation, pollution reduction, energy saving and resource sustainability. However, its real-life applications are limited by a lack of effective strategy to realize multicolour and water-jet printing on rewritable paper with long legible image-lasting times. Herein, we report an effective strategy to construct rewritable paper based on colour or luminescence switching induced by dynamic metal–ligand coordination. This type of rewritable paper can be conveniently utilized for multicolour water-jet printing by using aqueous solutions containing different metal salts as ink. In addition, the printed images on the water-jet rewritable paper can be retained for a long time (> 6 months), which shows great progress compared to previous work. We believe that this type of rewritable paper could be considered as a prototype for multicolour water-jet printing to meet the practical needs.

## Abstract (web summary)

Rewritable paper is environmentally favourable, but its practical realization is stifled by limited ink colour versatility and poor image retention times. Here, the authors exploit the relatively stable but reversible nature of metal–organic coordination bonds to produce long-lasting and multicoloured inks for rewritable paper.

## Introduction

For centuries, paper has been the most important media for mankind to record information and spread civilization. Although our daily lives have been filled with various electronic media in the past two decades, paper still occupies a crucial position in communication and information dissemination and storage. However, most of the paper can only serve as a disposable recording medium, which not only raises the cost but also causes numerous problematic issues, such as deforestation, solid waste, environmental pollution, energy consumption and so on^1^. Rewritable paper, which can be reused for multiple times, is therefore an attractive alternative that has economical and environmental benefits to human society^2–5^.

Stimuli-responsive photofunctional materials, which show reversible changes in optical properties responsive to external environment, are progressively emerging and have shown potential in the application of rewritable paper. To date, progress has been made in this research field. Several external-stimuli (such as humidity, light and hydrogen peroxide, etc.) responsive materials exhibiting colour or luminescence switching have been developed for fabrication of rewritable paper^6–16^. For instance, Zhang and colleagues^8^ demonstrated a novel approach for constructing water-jet rewritable paper that can be integrated into commercial desktop printer. In this rewritable paper, colourless oxazolidine-based hydrochromic dyes were incorporated, which undergo ring-opening reactions in the presence of water accompanied by the appearance of blue colour. Yin and colleagues^9^ have demonstrated a photoreversible colour switching system based on the redox dyes in response to photocatalytic reactions of TiO_2_ nanoparticles using ultraviolet light. Information can be efficiently printed on the rewritable paper containing these photochromic dyes under ultraviolet irradiation and erased by heat. Current research efforts are focused on the development of the rewritable papers with multicolour display and long recording time to improve their practical applications. In addition, low toxicity and cost of rewritable paper are also very important for daily use. Therefore, it is of great significance to develop new types of rewritable paper with the above-mentioned advantages.

Here, we describe a strategy that takes advantage of the reversibility of dynamic metal–ligand interactions to achieve rewritable paper and address the existing limits. In this work, we report the coating of a polymer film containing terpyridine ligand on filter paper on which multicolour images can be repeatedly painted using different metal salt aqueous solutions (MSAS) as the inks, which can coordinate with ligand. The printed pictures can be retained for over 6 months and erased conveniently by tetrabutylammonium fluoride (TBAF) to dissociate the metal–ligand coordination bond. Moreover, another type of rewritable paper, which can record information by using pure water as the ink, has been fabricated by incorporating water-responsive luminescent zinc complex into the imaging layer. As water is a green resource and has no risk to the environment, it is of great significance to develop water-jet rewritable paper. Our results demonstrate that the application of dynamic metal–ligand coordination in the preparation of rewritable paper would be a promising approach for the achievement of multicolour water-jet printing with long retention time.

## Results

### Design and synthesis

**Fig. 1:**
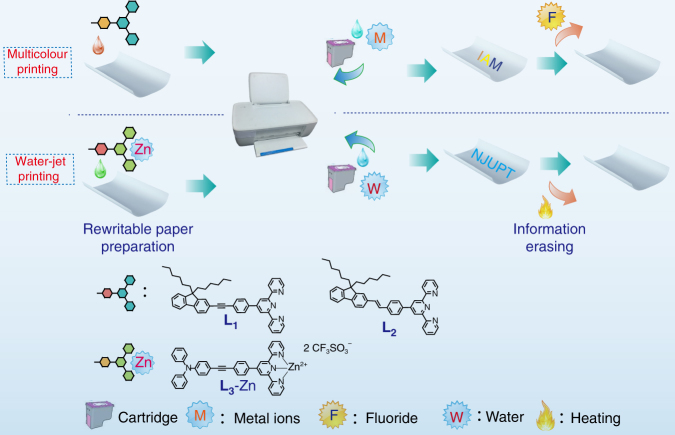
Schematic illustration of multicolour and water printing. Chemical structures of **L**_**1**_, **L**_**2**_ and **L**_**3**_, and the printing and erasing processes for the constructed rewritable paper

In our design, the interaction and dissociation of dynamic metal–ligand coordination bonds are the basic reactions that occur in printing and erasing (see Fig. 1). The dynamic metal–ligand coordination bonds are one important kind of supramolecular interactions, which is similar to hydrogen bond with intermediate strength and good reversibility^17–24^. The stimuli-responsive materials based on dynamic metal–ligand coordination possess following advantages: the metal–ligand coordination bonds are stable under ambient conditions, and therefore their colour or luminescence change can keep for a long period, the reversible reaction conditions for dynamic metal–ligand coordination bonds are simple and convenient and various colours or luminescence can be achieved when the ligands are coordinated to different metal ions. Therefore, we conjectured that the construction of multicolour rewritable paper through dynamic metal–ligand interaction would be a promising strategy.

Tridendate ligands, such as terpyridine derivatives, are capable of forming ligand–metal complexes with a variety of metal ions^25–27^. Our molecular design is based on the synthesis of terpyridine compounds with donor–π–acceptor structure, which could exhibit intense absorbance and different colours. This feature could make it possible for the fabricated rewritable paper to generate more colours. Here, three terpyridine derivatives (**L**_**1**_, **L**_**2**_ and **L**_**3**_) have been designed and prepared as examples to demonstrate the feasibility of metal–ligand coordination-based rewritable paper (Fig. 1). The fluorene or triphenylamine group conjugated to the phenyl ring was chosen as the electron donor and the terpyridine group was the electron acceptor. The detailed synthetic procedures have been shown in the Supplementary Methods. The obtained compounds were characterized by ^1^H and ^13^C nuclear magnetic resonance spectroscopy (NMR) spectroscopy, and matrix assisted laser desorption ionization-time of flight (MALDI-TOF) mass spectrometer (MS).

### Multicolour printing

**Fig. 2:**
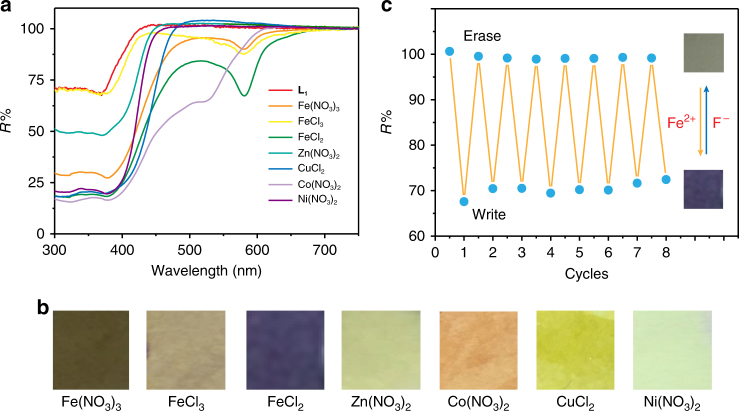
Multicolour and reversibility of rewritable paper. **a** The reflective UV–visible spectra of rewritable paper constructed with **L**_**1**_ after addition of Fe(NO_3_)_3_, FeCl_3_, FeCl_2_, Ni(NO_3_)_2_, Co(NO_3_)_2_, CuCl_2_ and Zn(NO_3_)_2_, respectively. **b** The photographs of rewritable paper produced by addition of different metal salts. **c** A plot of the reflectivity at 577 nm vs. the number of cycles as the rewritable paper is cycled through FeCl_2_ aqueous solution spraying (writing) and rinsing the rewritable paper with CH_2_Cl_2_ solution of TBAF (erasing)

We take **L**_**1**_ as an example herein to demonstrate the absorption variations caused by the formation of metal–ligand coordination. Quick solution tests of **L**_**1**_ coordinated with different metal ions (Fe^3+^, Fe^2+^, Ni^2+^, Co^2+^, Cu^2+^ and Zn^2+^) were conducted firstly in CH_2_Cl_2_ solution, which reveal dramatic changes in absorption bands. As shown in Supplementary Fig. 1, upon addition of Fe^2+^, a new absorption band of **L**_**1**_ in CH_2_Cl_2_ emerged at 575 nm, which is assignable to the metal-to-ligand charge-transfer (MLCT) transition that is characteristic of the Fe^2+^ complexes with π-accepting terpyridine ligands. After the coordination between the Co^2+^/Fe^3+^ and **L**_**1**_, the new absorption bands corresponding to either the *d–d* transitions or MLCT band were raised (see Supplementary Figs. 2–4). When **L**_**1**_ chelated with Ni^2+^ and Cu^2+^, *π*–π* transitions or *d*–*d* transitions are responsible for the changes in the absorption spectra (see Supplementary Figs. 5 and 6). As shown in Supplementary Fig. 7, the appearance of new absorption peaks when **L**_**1**_ chelated with Zn^2+^ can be attributed to the intraligand charge-transfer transitions^28^. Thus, the resulted complexes exhibited seven distinguishable colours when **L**_**1**_ was coordinated with Fe(NO_3_)_3_, FeCl_3_, FeCl_2_, Ni(NO_3_)_2_, Co(NO_3_)_2_, CuCl_2_ and Zn(NO_3_)_2_, respectively (see Fig. 2). To achieve the rewritable paper, one critical issue is the good reversibility between two colour states. It is known that the fluoride ions tend to coordinate with hard metal ions, such as Fe^3+^, Fe^2+^, Zn^2+^, Ni^2+^, Co^2+^ and Cu^2+^, and it has been demonstrated that the interactions between various metal ions and fluoride would dissociate the metal–ligand coordination^27, 29^. Thus, the UV–visible (UV–Vis) absorption spectra variations have been recorded by the addition of TBAF. When fluoride was added, the absorption bands were restored to original position (see Supplementary Figs. 8–14), resulting in the colour recovery. The MS spectra showed that the metal–ligand coordination bond has been cut off upon addition of TBAF (see Supplementary Fig. 15). This dissociation can be attributed to the stronger binding affinity between fluoride and metal ion than the metal–ligand interaction^27, 29^.

**Fig. 3:**
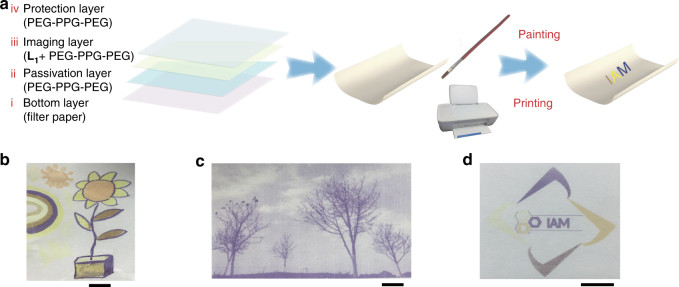
Structure and multicolour printing of rewritable paper. **a** Schematic illustrations of four-layer structure used to create the rewritable paper based on **L**_**1**_. **b** Colourful image of a flower drawn by different metal salts aqueous solution as ink. Scale bar = 1 cm. **c** An image of trees printed using a customized black inkjet cartridge filled with FeCl_2_ aqueous solution. Scale bar = 1 cm. **d** Colourful printing of the badge of Institute of Advanced Materials using an inkjet cartridge filled with FeCl_2_, Zn(NO_3_)_2_ and Co(NO_3_)_2_, respectively. Scale bar = 1 cm

Having achieved multicolour in the solution, we expected that it can also be realized on the paper substrate. Hence, a four-layer structure of rewritable paper has been developed using poly(ethylene glycol)-*block*-poly(propylene glycol)-*block*-poly(ethylene glycol) (PEG-PPG-PEG) to enhance its practical usability (see Fig. 3a). PEG-PPG-PEG possesses good swell ability for water, which facilitates the penetration of MSAS, and consequently increases the opportunity of metal ions coordination with ligand. The four layers of the rewritable paper structure include: the bottom layer being filter paper as a substrate, a passivation layer consisting of PEG-PPG-PEG directly in contact with the paper substrate as an isolation belt between ligand and paper surface, a thin film of PEG-PPG-PEG and the ligand serving as the imaging layer and a final layer of PEG-PPG-PEG coated on the surface acting as a protection layer. The original colour of the rewritable paper is consistent with the colour of filter paper because the PEG-PPG-PEG+**L**_**1**_ is colourless (see Supplementary Fig. 16). Reflective UV–Vis spectroscopy was used to measure the produced rewritable paper upon addition of different MSAS (see Fig. 2a). As shown in Fig. 2b, seven distinguishable colours including brown, khaki, deep blue, pale yellow, orange, yellow and light green were obtained on our constructed rewritable paper. Moreover, it is found that when Fe^2+^ coordinated with **L**_**3**_, the black colour can be obtained as shown in Supplementary Fig. 17. This finding suggested that by modifying the chemical structure of the ligand, more colours can be developed. It is believed that by preparing and screening the ligands with different chemical structures, the achievement of full colour displays is possible. Next, the reversibility and repeatability on the rewritable paper were investigated. For example, in the absence of Fe^2+^, the rewritable paper exhibits an absorption band centred on 376 nm, which is the characteristic feature of **L**_**1**_. Upon addition of aqueous solution of Fe^2+^, a new absorption band centred at 577 nm was observed. This colour can be erased by immersing the rewritable paper into CH_2_Cl_2_ solution of TBAF, and it took about 5 min to fade the colour. The reflectivity at 577 nm was recorded after repetitive writing with Fe^2+^ aqueous solution and erasing by CH_2_Cl_2_ solution of fluoride. Only a slight decrease in colour intensity was observed after more than 5 consecutive write–erase cycles (see Fig. 2c), indicating the feasibility of our produced paper for multiple-time use.

The application of freehand writing on our rewritable paper has been developed first. Using a pen with FeCl_2_ solution as ink, one sentence has been handwritten on the rewritable paper as shown in Supplementary Fig. 18. Then, by using a commercially available inkjet printer with a cartridge filled with MSAS, images have been successfully printed on the rewritable paper. For example, good resolution of deep-blue image of trees has been achieved by our water-jet printing system using a small amount of FeCl_2_ aqueous solution as ink (see Fig. 3c). Moreover, the legibility of the printed picture can last at least over 6 months under ambient conditions (see Supplementary Fig. 19), which is much longer than that of currently developed rewritable papers^8–12^. Furthermore, by using various MSAS as inks, multicolour printing has been achieved on our rewritable paper. As shown in Fig. 3b, the image of a flower exhibited different and distinguishable colours when applying different MSAS painting on the rewritable paper. Moreover, by loading aqueous solutions of FeCl_2_, Zn(NO_3_)_2_ and Co(NO_3_)_2_ into a tri-colour inkjet cartridge, a colourful image of the badge of Institute of Advanced Materials has been successfully printed on the rewritable paper (see Fig. 3d).

The recolouration is a very important issue for the rewritable paper. Supplementary Fig. 20 shows that there is no detectable decrease in colour intensity of the written numbers by naked eyes, indicating good recolouration ability of the rewritable paper. Furthermore, the influence of TBAF solution adsorbed in the paper on recolouration was investigated by the reflective UV–Vis spectra. The spectra were recorded on the rewritable paper with and without F^−^ treatment after addition of FeCl_2_ aqueous, and only a slight decrease in reflective UV–Vis spectra was observed for the F^−^-treated rewritable paper (see Supplementary Fig. 21).

### Luminescence printing

The photoluminescence (PL) spectra variations of these terpyridine derivatives induced by the coordination with various metal ions have also attracted our attention. The development of multicolour luminescence printing is an important topic since it is often associated with information encryption technique^30–34^. Herein, the PL spectra changes of **L**_**1**_ by coordination with different metal ions were studied. The emission of **L**_**1**_ at 423 nm in CH_2_Cl_2_ was quenched significantly by the addition of different metal ions (Fe^3+^, Fe^2+^, Ni^2+^, Co^2+^ and Cu^2+^) except for Zn^2+^ (see Supplementary Fig. 22). In the case of zinc ion, the emission band at 423 nm was quenched with a newly emerged band at a longer wavelength. The bathochromic-shifted emissions of **L**_**1**_ **-Zn** were strongly influenced by the counterion in the respective metal salts (see Supplementary Fig. 23). For example, the emission peak of **L**_**1**_ in CH_2_Cl_2_ (*λ*_em_ = 423 nm) was red-shifted to 494 nm upon coordinating with Zn(CH_3_COO)_2_. In the case of Zn(NO_3_)_2_ and Zn(CF_3_SO_3_)_2_, the emission peak located at 509 and 522 nm, respectively. Unfortunately, the emission wavelength tuning range of **L**_**1**_ by different zinc salts is only about 28 nm, which is too narrow to exhibit separate emission colours. To address this limit, ligand **L**_**2**_ was designed and prepared. For **L**_**2**_, the ethylene bond was introduced to replace the acetylene bond of **L**_**1**_. The introduction of ethylene bond into the ligand results in a higher degree of the π-electron delocalization of the whole molecular system. Thus, the luminescence property of **L**_**2**_ is more easily affected by the external environment.

**Fig. 4:**
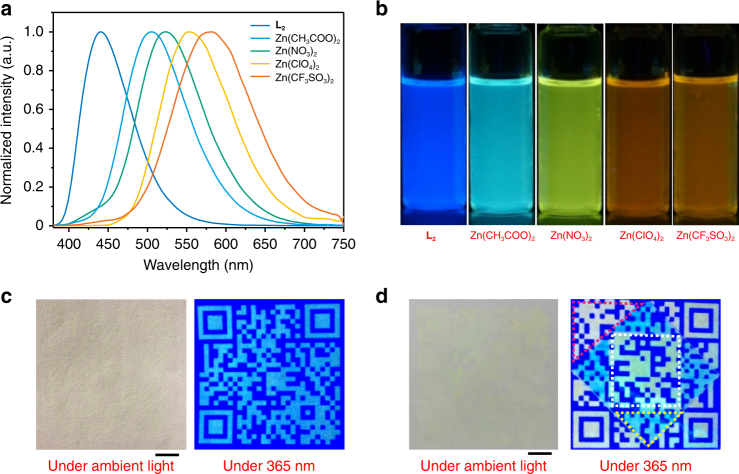
Photoluminescent properties and security printing. **a** Room-temperature normalized photoluminescence spectra of **L**_**2**_ and Zn(II) complexes in CH_2_Cl_2_ (10 μM). **b** Photographs of the emission colours of **L**_**2**_ and Zn(II) complexes in CH_2_Cl_2_. **c** An image of QR code under daylight and UV light printed using a customized black inkjet cartridge filled with Zn(CH_3_COO)_2_ aqueous solution. **d** A colourful image of QR code under ambient light and UV light printed using a customized tri-colour inkjet cartridge filled with Zn(CH_3_COO)_2_, Zn(NO_3_)_2_ and Zn(CF_3_SO_3_)_2_ aqueous solution. Scale bar = 1 cm

Next, the PL spectra changes of **L**_**2**_ by coordination with different metal ions were investigated. The emission peak (*λ*_em_ = 441 nm) of **L**_**2**_ in CH_2_Cl_2_ was red-shifted to 505, 523, 553 and 581 nm upon coordinating with Zn(CH_3_COO)_2_, Zn(NO_3_)_2_, Zn(ClO_4_)_2_ and Zn(CF_3_SO_3_)_2_, respectively (see Fig. 4a and Supplementary Figs. 24–27). Thus, the wider emission wavelength tuning range has been achieved, and the emission colours of cyan, green, yellow and orange were realized. Our previous study has demonstrated that this interesting change in luminescence colour was attributed to the difference in basicity of various counterions^33^. The decrease of the basicity of counterions would stabilize the LUMO energy of the coordinated zinc complexes, resulting in the red shift of emission band. This metal–ligand coordination bond can also be dissociated upon addition of TBAF to the CH_2_Cl_2_ solution (see Supplementary Figs. 28–31). The proton nuclear magnetic resonance (^1^H NMR) technique was employed to confirm the coordination/dissociation processes between Zn^2+^ and **L**_**2**_. Supplementary Fig. 32 shows the change of chemical shifts of **L**_**2**_ upon addition of 1 equiv. of Zn^2+^. The resonance signals of protons on the terpyridine ligand exhibit an obvious upfield shift, which was generated by the coordination between Zn^2+^ and **L**_**2**_. After the addition of 10 equiv. F^−^ into the solution containing **L**_**2**_ **-**Zn, the chemical shifts of protons on the terpyridine ligand were recovered completely.

After integration of **L**_**2**_ on the filter paper substrate using similar procedures to **L**_**1**_, high-resolution QR code can be clearly observed under UV light by using Zn(CH_3_COO)_2_ aqueous solution as ink (see Fig. 4c), and the images can be lasting for over 1 year (see Supplementary Fig. 33). In sharp contrast, the produced QR code is almost invisible to the naked eyes under natural light. This feature is ideal for applications in security printing technologies. Significantly, when one QR code consists of different emission colours, it would be more difficult to counterfeit. In addition, it could greatly improve the security of documents and enlarge the current amount of data storage. Therefore, we have loaded aqueous solutions of Zn(CH_3_COO)_2_, Zn(NO_3_)_2_ and Zn(CF_3_SO_3_)_2_ into tri-colour inkjet cartridge and successful printed multicolour luminescent QR code. Furthermore, we have tried to print a microscale pattern on the rewritable paper. As shown in the confocal images in Supplementary Fig. 34, microscale patterns of thin line and dots line with the width around 200 μm could be successfully printed on the rewritable paper, indicating an attainable resolution. The result is comparable to the patterns printed by normal commercial inkjet printer (HP Desk Jet 1110) on A4 paper (see Supplementary Fig. 34). The resolution of printing is also determined by the size of the inkjet nozzle of the printer we used. Hence, it is believed that the higher resolution images might be obtained on our rewritable paper by employing a high precision printer. These images can be erased by immersing the rewritable paper into CH_2_Cl_2_ solution of TBAF as well, and the information vanished within 5 min (see Supplementary Fig. 35).

### Water-jet printing

**Fig. 5:**
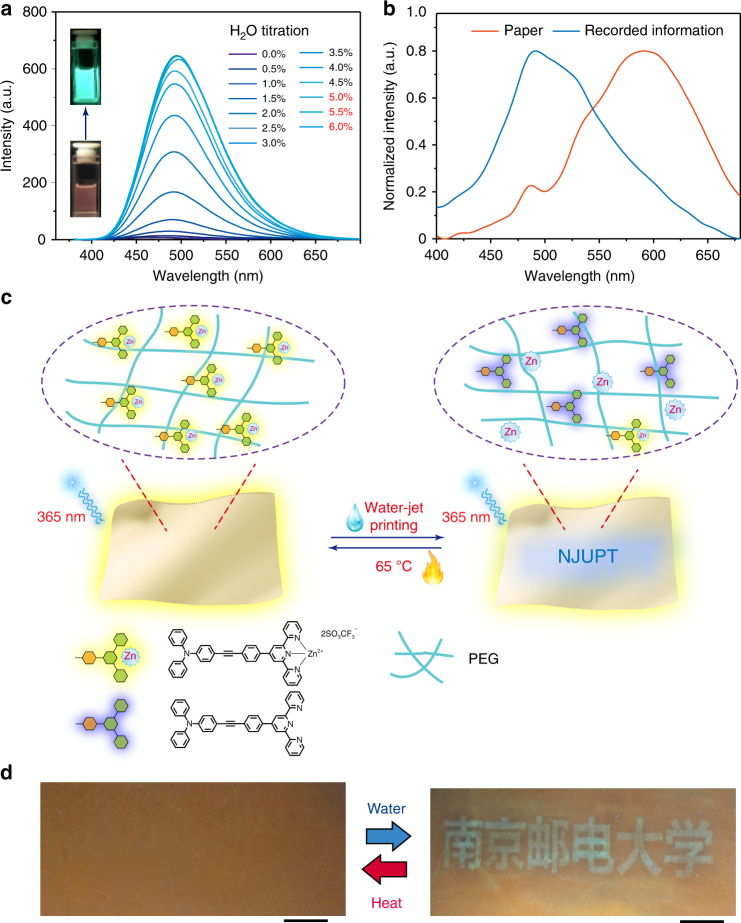
Water-jet printing of rewritable paper. **a** PL spectra of complex **L**_**3**_ **-Zn** in THF–water mixtures with different water fractions. **b** The emission spectra of **L**_**3**_ **-Zn** before and after applying water on the rewritable paper. **c** Schematic illustrations of the writing and erasing processes of water-jet printing of rewritable paper based on **L**_**3**_ **-Zn**. **d** Water-jet prints by a commercially available inkjet printer with cartridges refilled with water on the rewritable paper based on **L**_**3**_ **-Zn**. Scale bar = 1 cm

From a green perspective, it would be ideal if pure water can stimulate luminescence switches for the development of rewritable paper. It is known that the dynamic metal–ligand coordination bonds undergo reversible dissociation/coordination^34, 35^. For zinc complex, in the presence of polar solvents, there would be a chemical equilibrium arising from the dissociation/coordination between zinc complex and ligand+zinc salts^36^, thus resulting in the luminescence change. Therefore, the PL spectra change of zinc complex by addition of small amount of pure water was investigated. First, **L**_**2**_ was mixed with 1 equiv. Zn(CF_3_SO_3_)_2_ in tetrahydrofuran (THF) solution for the formation of zinc complex (**Zn-L**_**2**_) (see Supplementary Fig. 36). The PL spectrum of **Zn-L**_**2**_ was then recorded in THF with increasing the water portion (see Supplementary Fig. 37). It was observed evidently that the long-wavelength emission (542 nm) was gradually quenched, but a new emission (441 nm) emerged. When the water content increased to 5%, the new emission kept unchanged. The relative ratio of luminescence intensities (*I*_441 nm_/*I*_542 nm_) increases 47-fold (from 0.005 to 2.350) over the water content range of 0–5% (see Supplementary Fig. 38). To achieve the satisfied water-jet rewritable paper, the high contrast is quite crucial. Hence, ligand **L**_**3**_ was prepared by attaching a stronger donor group of triphenylamine on the terpyridine to improve the contrast ratio of luminescence variation of zinc complex by the addition of pure water. The PL spectral change of **Zn-L**_**3**_ in THF solution by the addition of water was recorded (see Fig. 5a), and the emission intensity at 498 nm enhanced dramatically with the increase of water portion. The relative ratio of luminescence intensities (*I*_631 nm_/*I*_498 nm_) increases by 97-fold (from 1.1 to 106.9) over the water content range of 0–5%, indicating a higher contrast ratio than that of **Zn-L**_**2**_. Besides, the PL spectra of **Zn-L**_**3**_ in THF at different concentrations with 5% content water was investigated (see Supplementary Fig. 39). As the concentration increases from 1 × 10^−5^ to 1 × 10^−2^ M, the emission intensity at 498 nm gradually disappears, suggesting that this water-induced luminescence change can only be observed in dilute solution. These observations suggest that **Zn-L**_**3**_ is an ideal candidate for the construction of water-jet rewritable paper.

Then, the rewritable paper has been fabricated by the same procedure as illustrated above, except that the imaging layer was replaced by **Zn-L**_**3**_ and the polymer matrix was changed to PEG. The reason why the PEG-PPG-PEG was replaced is that PEG shows better swell ability for water and lower melting point. It is expected that water can permeate through the PEG protective layer and then dissociate the coordination bond. Figure 5c shows that the fabricated rewritable paper was successfully utilized for printing luminescent characters by a commercially available inkjet printer with a cartridge filled with pure water. The paper exhibits orange luminescence under UV light. Chinese characters of 'Nanjing University of Posts and Telecommunications' were obtained by combining water-jet printing. In the daylight, the printed information is almost invisible on the paper. The hidden characters on this paper are observable under UV light with cyan luminescence. For the previous report about rewritable paper for water-jet printing, the information would be faded after the evaporation of water^8^. Unexpectedly, herein, the information recorded on **L**_**3**_ **-Zn**-based rewritable paper can be kept even after the water evaporation, and the legible emission can last over 6 months under ambient condition (see Supplementary Fig. 40). This might be attributed to that PEG can act as a solid-state solvent and prevent the interaction between zinc ion and ligand again after the dissociation. However, the recorded information can be erased by mild heating, and it takes about 30 min to vanish the information at 65 °C. The melting point of PEG applied in this work is ranged from 64 to 66 °C. Hence, PEG would change its solid state to melt state so that the zinc ions have the opportunity to coordinate with ligand, thus leading to luminescence recovery. Our water-jet rewritable paper can retain information for a long period (over 6 months), which greatly improves its practical applications.

## Discussion

In summary, we present an effective strategy to develop ink-free rewritable paper by introducing **L**_**1**_, **L**_**2**_ and **L**_**3**_ **-Zn** in paper and using MSAS and even pure water as the trigger. Important insights on the relationship between metal ions and terpyridine ligand with their photophysical properties have been obtained, which allows us to realize multicolour information recording with long image lifetime on one rewritable paper. Moreover, having gained insights on the water-triggered dynamic metal–ligand coordination bond dissociation, water-jet rewritable paper has also been achieved. The rewritable paper with legibility lasting over 6 months per print under ambient conditions has been developed, and numbers of write–erase cycles without obvious colour or luminescence fading have been realized.

Furthermore, according to a rough estimation, the cost per print of the multicolour rewritable printing and water-jet rewritable printing (based on a conservative 8 times reusage/sheet) would be approximately one-fifth and one-tenth of the normal inkjet print (see Supplementary Tables 1 and 2), indicating the low cost of this rewritable printing system. Moreover, the cytotoxicity measurements of the terpyridine ligands and different metal complexes we used in this work were performed by using the standard methyl thiazolyl tetrazolium (MTT) assays. Supplementary Table 3 shows cellular viabilities after different concentrations of the ligands and complexes were treated to cells for 24 h. The results showed that the cellular viabilities were assessed to be >75% even at a high concentration of 50 μM, which indicated that the ligands and complexes we used here were within low toxicity range. Besides, these rewritable materials are isolated by the PEG-PPG-PEG/PEG (well-known harmless materials) protective layer, which can further enhance the safety of the rewritable paper.

Overall, this type of rewritable paper is a promising candidate to address the growing problems in resource sustainability and environment. Particularly, the elaborate features of multicolour printing on the same page with long retention time greatly improve the practical usability of our rewritable paper. It is expected that our design principle can be extended to various dynamic supramolecular interactions to produce advanced rewritable paper and green printing technology.

## Methods

### Materials

Unless otherwise stated, all starting materials and reagents were purchased from commercial suppliers and used without further purification. All solvents were purified before use. The solvents were carefully dried and distilled from appropriate drying agents prior to use.

### Measurements

^1^H NMR (400 MHz) and ^13^C NMR (100 MHz) spectra were recorded on a Bruker ACF400 spectrometer at 298 K using deuterated solvents. The ^1^H NMR chemical shifts are reported relative to tetramethylsilane (TMS) (0.00 ppm) or residual protonated solvents (7.26 ppm for CDCl_3_ or 1.72, 3.58 ppm for THF-*d*_*8*_). The ^13^C NMR chemical shifts are reported relative to TMS (0.00 ppm) or deuterated solvents (77.0 ppm for CDCl_3_). Mass spectra were recorded on a Bruker autoflex MALDI-TOF MS. The UV–Vis absorption spectra were obtained with a Shimadzu UV-3600 UV–VIS–NIR spectrophotometer. Reflection spectroscopy was measured with a Shimadzu UV-2600 UV–VIS–NIR. *X*-axis shows the light wavelength, and *y*-axis represents reflectivity. PL spectra were measured with HITACHI F-7000 fluorescence spectrophotometer.

### Preparation of rewritable paper

The rewritable paper integrated with **L**_**1**_ or **L**_**2**_ was prepared in a layer-by-layer manner. First, the filter paper substrate was coated with a passivation layer of PPG-PEG-PPG (95 mg/mL, average *M*_n_: ~ 14,600) CH_2_Cl_2_ solution by a brush and dried at 40 °C in vacuum. Second, the imaging layer was constructed by painting CH_2_Cl_2_ solution of **L**_**1**_ or **L**_**2**_ (3 mg/mL) and PPG-PEG-PPG (95 mg/mL) on the passivation layer and then dried at 40 °C in vacuum. Last, another protective layer of PPG-PEG-PPG was coated on the top by using a brush. The fabrication of water-jet rewritable paper can follow same procedure as illustrated above, except that the imaging layer was replaced by **Zn-L**_**3**_ and the polymer matrix was changed to PEG (average *M*_n_: ~ 20,000).

### MTT assays

In vitro cytotoxicity was measured by performing MTT assays on HeLa cells. Cells were seeded into a 96-well cell culture plate at 104/well, under 100% humidity, and were cultured at 37 °C with 5% CO_2_ for 24 h. Different concentrations (1, 5, 10 and 50 μM) of rewritable materials (**L**_**1**_, **L**_**2**_, **L**_**3**_, **L**_**1**_ **-Fe(NO**_**3**_)_**3**_, **L**_**1**_ **-FeCl**_**3**_, **L**_**1**_ **-FeCl**_2_, **L**_**1**_ **-Co(NO**_**3**_)_**2**_, **L**_**1**_ **-CuCl**_**2**_, **L**_**1**_ **-Ni(NO**_**3**_)_**2**_, **L**_**1**_ **-Zn(NO**_**3**_)_**2**_, **L**_**2**_ **-Zn(CH**_**3**_ **COO**)_**2**_, **L**_**2**_ **-Zn(NO**_**3**_)_**2**_, **L**_**2**_ **-Zn(ClO**_**4**_)_**2**_, **L**_**2**_ **-Zn(CF**_**3**_ **SO**_**3**_)_**2**_ and **L**_**3**_ **-Zn(CF**_**3**_ **SO**_**3**_)_**2**_) were then added into the wells. The cells were subsequently incubated for 24 h at 37 °C under 5% CO_2_. Then, MTT (10 μL/well, 5 mg/mL) was added to each well and the plate was incubated for an additional 4 h at 37 °C under 5% CO_2_. The medium was then replaced with 150 μL dimethyl sulfoxide per well, and OD570 was monitored by an enzyme-linked immunosorbent assay reader. The following formula was used to calculate the inhibition of cell growth: Cell viability (%) = (mean of Abs. value of treatment group/mean Abs. value of control) × 100%.

### Data availability

The data supporting the findings of this study are available from the corresponding author on request.

## Electronic supplementary material

Supplementary Information
