# Supplementary material for: Dynamic metal-ligand coordination for multicolour and water-jet rewritable paper
Source: Nat Commun. 2018 Jan 9;9:3. doi: 10.1038/s41467-017-02452-w (PMC5760713; doi:10.1038/s41467-017-02452-w)
Supplement: Supplementary file 1 — Supplementary Information [file 41467_2017_2452_MOESM1_ESM.pdf]

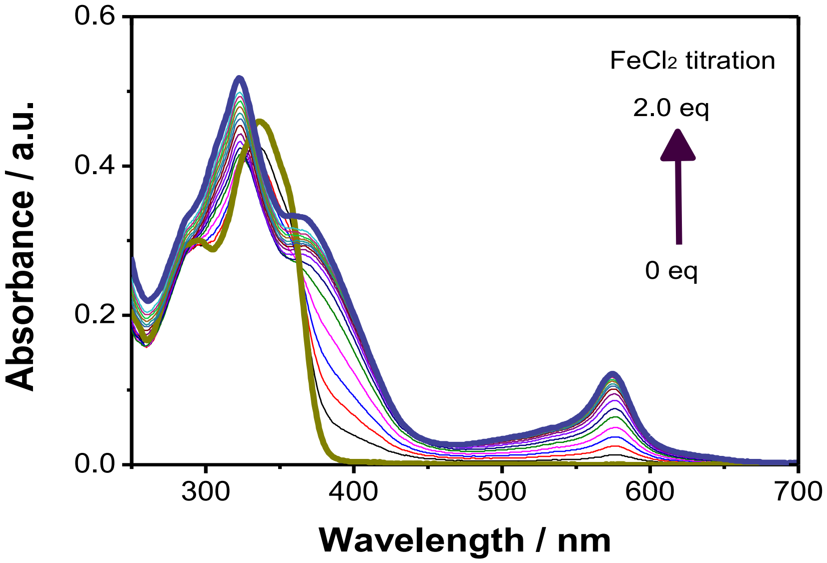

Supplementary Figure 1. Absorption spectral change of $\mathbf{L_1}$ with $FeCl_2$ titration in CH_2Cl_2/EtOH (v/v, 199/1) mixture (10 μM).

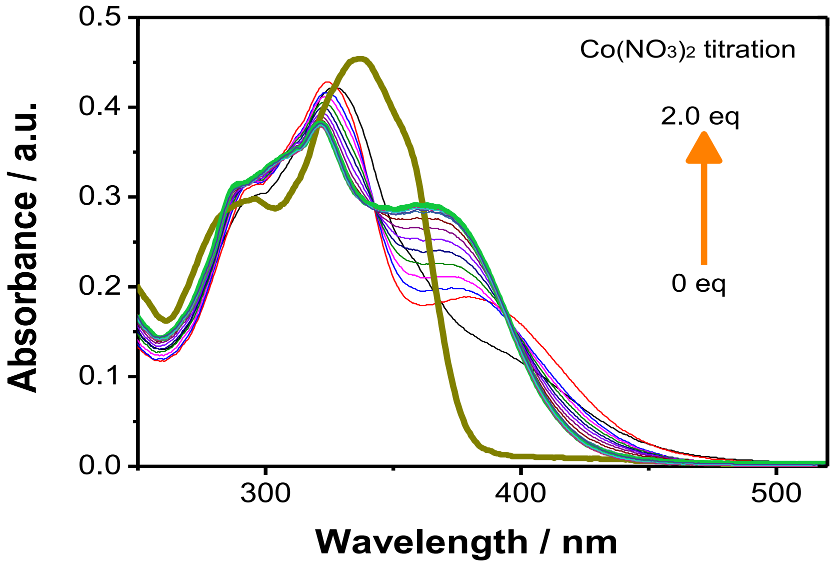

Supplementary Figure 2. Absorption spectral change of **L_1** with $Co(NO_3)_2$ titration in CH_2Cl_2/EtOH (v/v, 199/1) mixture (10 μM).

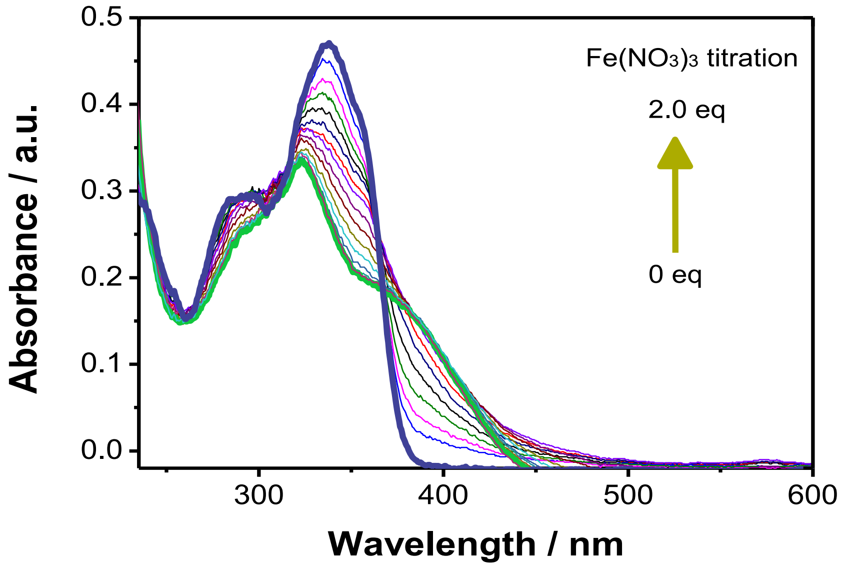

Supplementary Figure 3. Absorption spectral change of **L_1** with $Fe(NO_3)_3$ titration in CH_2Cl_2/EtOH (v/v, 199/1) mixture (10 μM).

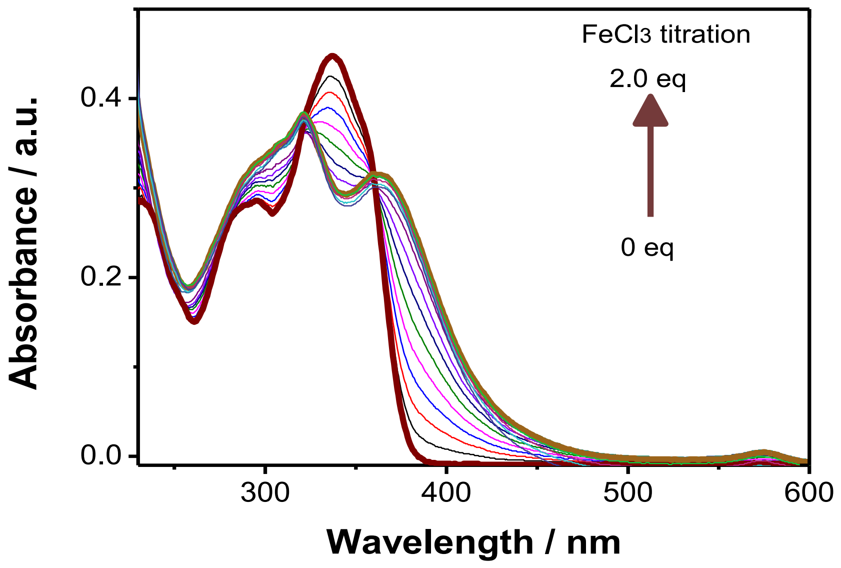

Supplementary Figure 4. Absorption spectral change of **L_1** with $FeCl_3$ titration in CH_2Cl_2/EtOH (v/v, 199/1) mixture (10 μM).

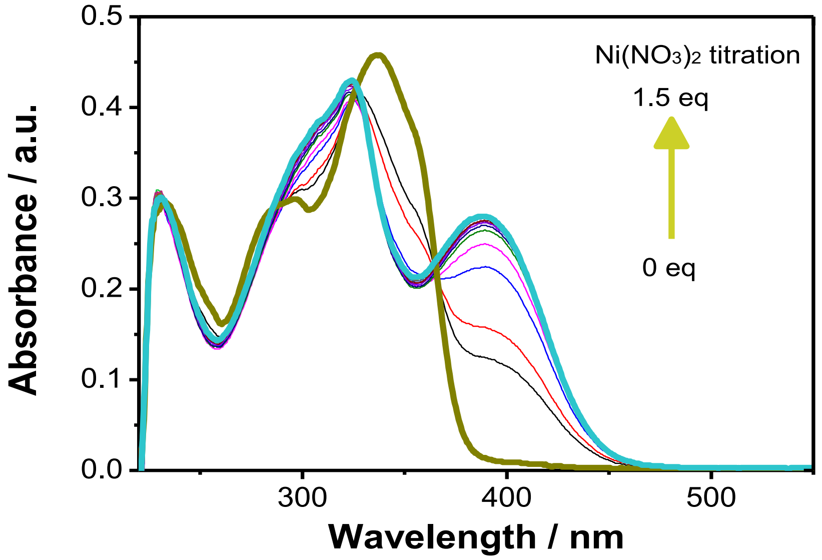

Supplementary Figure 5. Absorption spectral change of **L1** with $Ni(NO_3)_2$ titration in CH_2Cl_2/EtOH (v/v, 199/1) mixture (10 μM).

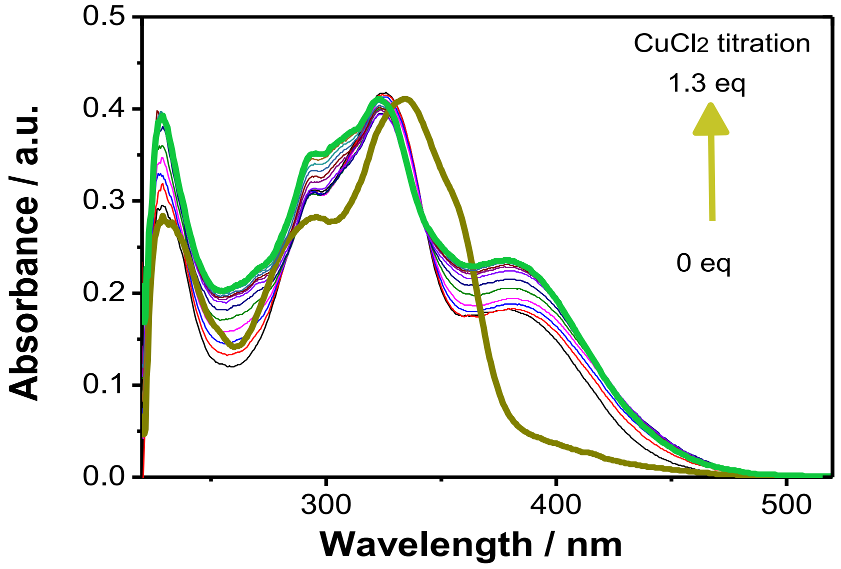

Supplementary Figure 6. Absorption spectral change of **L1** with $CuCl_2$ titration in CH_2Cl_2/EtOH (v/v, 199/1) mixture (10 μM).

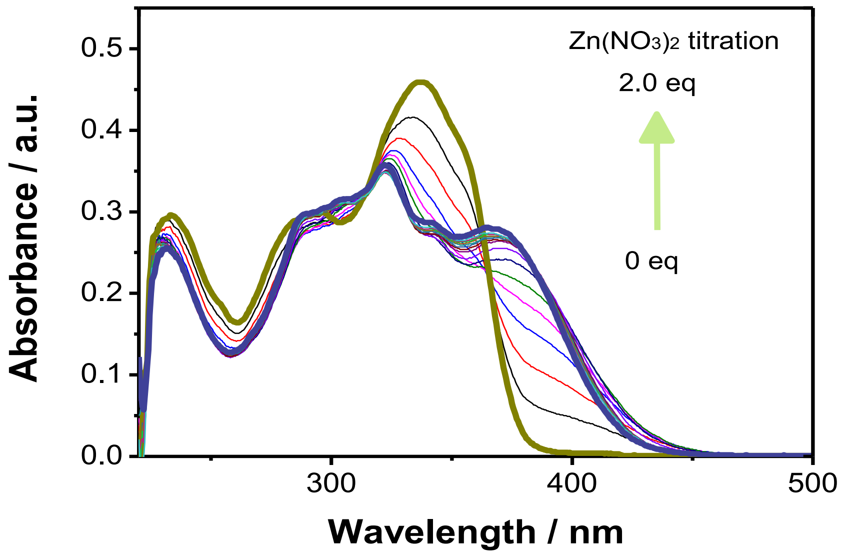

Supplementary Figure 7. Absorption spectral change of **L_1** with $Zn(NO_3)_2$ titration in CH_2Cl_2/EtOH (v/v, 199/1) mixture (10 μM).

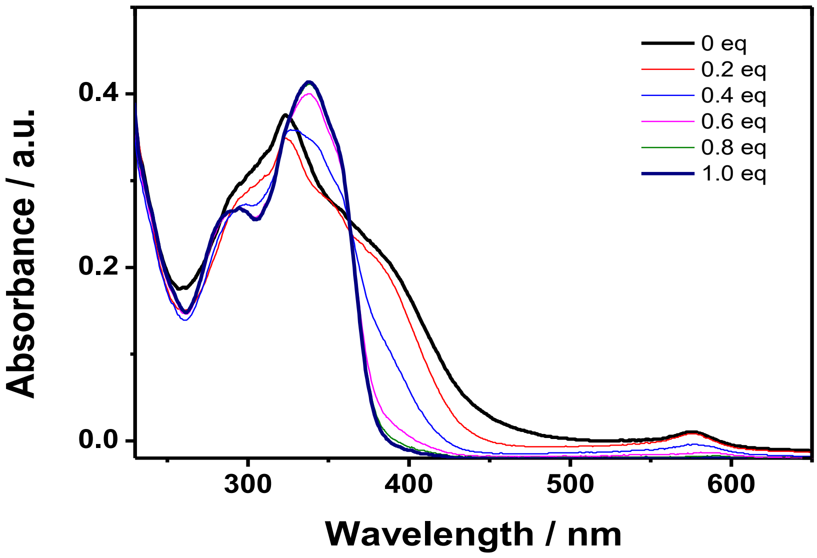

Supplementary Figure 8. Absorption spectral change of L_1-$Fe(NO_3)_3$ with F^- titration in CH_2Cl_2 solution (10 μM).

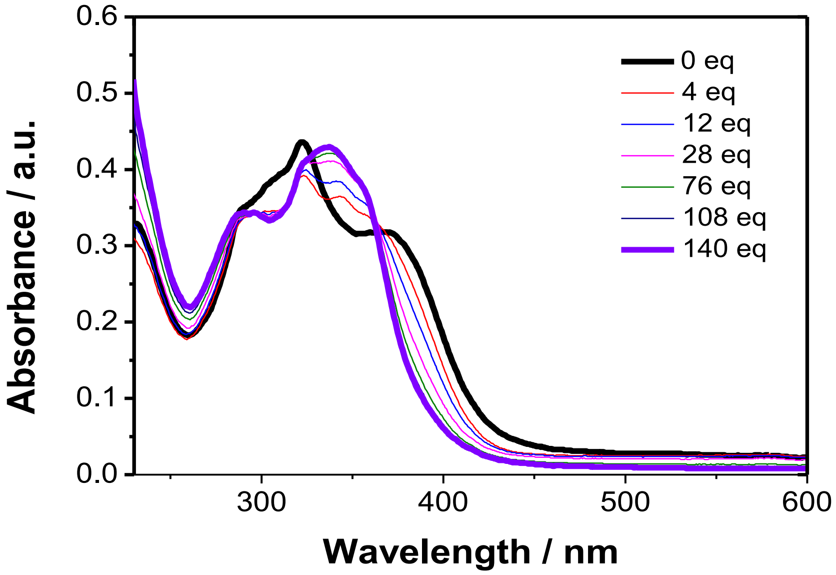

Supplementary Figure 9. Absorption spectral change of L_1-$Co(NO_3)_2$ with F^- titration in CH_2Cl_2 solution (10 μM).

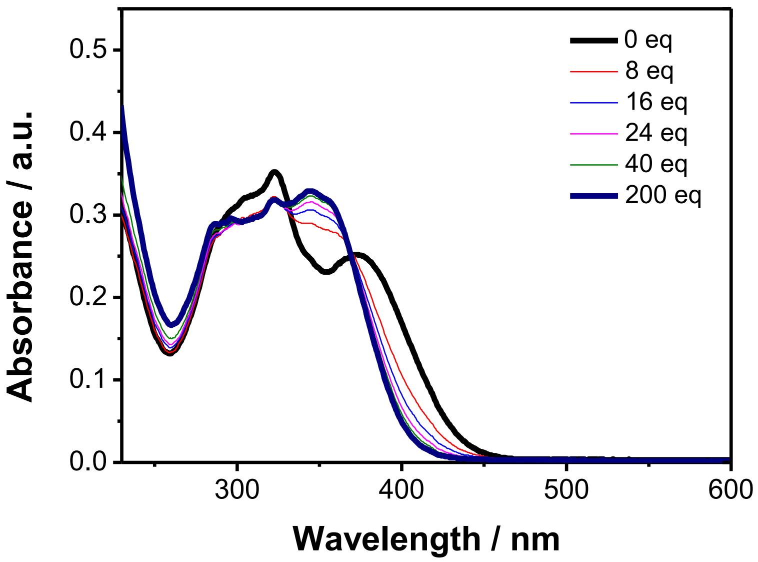

Supplementary Figure 10. Absorption spectral change of L_1-$Ni(NO_3)_2$ with F^- titration in CH_2Cl_2 solution (10 μM).

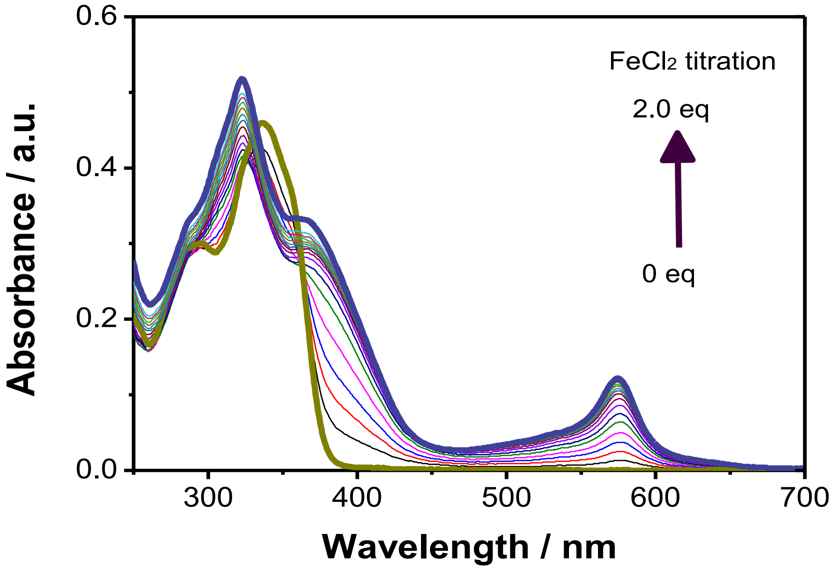

Supplementary Figure 11. Absorption spectral change of L_1-$FeCl_2$ with F^- titration in CH_2Cl_2 solution (10 μM).

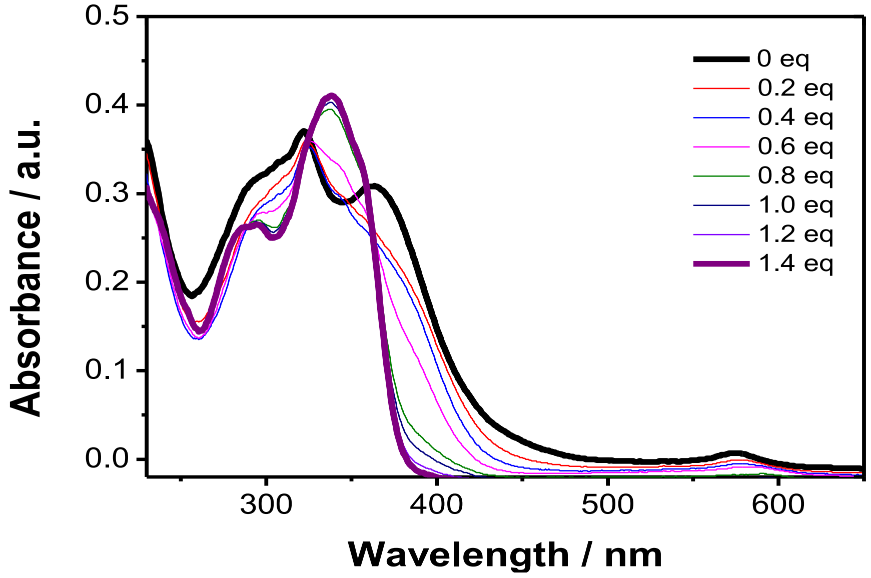

Supplementary Figure 12. Absorption spectral change of L_1-$FeCl_3$ with F^- titration in CH_2Cl_2 solution (10 μM).

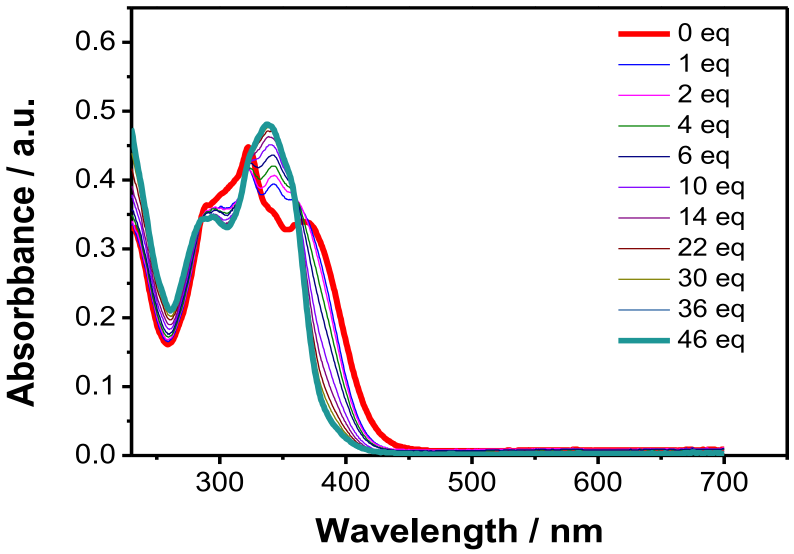

Supplementary Figure 13. Absorption spectral change of L_1-$Zn(NO_3)_2$ with F^- titration in CH_2Cl_2 solution (10 μM).

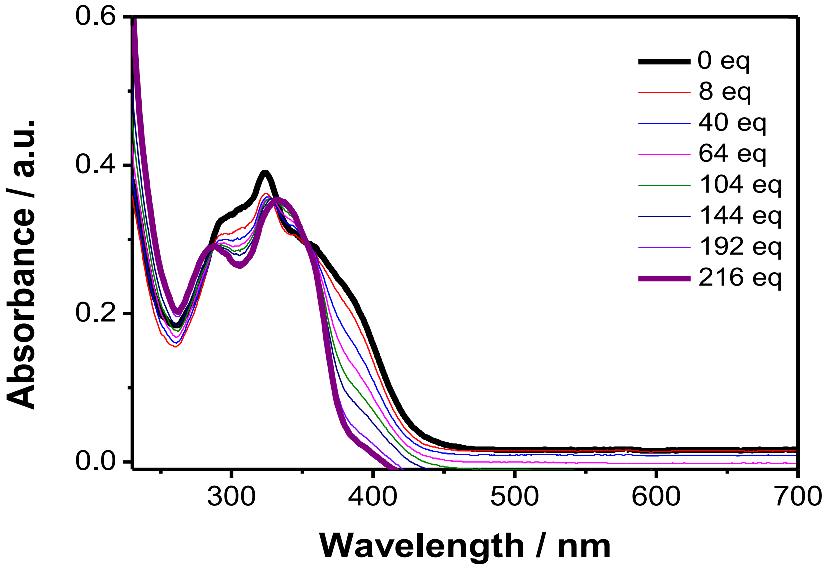

Supplementary Figure 14. Absorption spectral change of L_1-$CuCl_2$ with F^- titration in CH_2Cl_2 solution (10 μM).

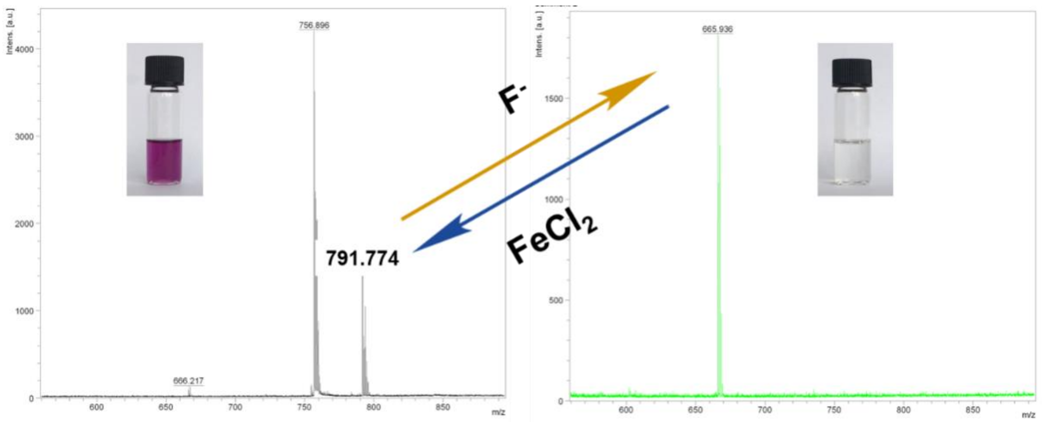

Supplementary Figure 15. The photographs and mass spectra of **L_1-$FeCl_2$** in CH_2Cl_2 (7.5×10^{-4} μM) before and after the addition of 2 equiv. $Bu_4N^+F^-$. calcd. for L_1, 665.93; found: 665.936; L_1-$FeCl_2$, 792.67; found: 791.774; L_1-$FeCl^+$, 757.22; found: 756.896.

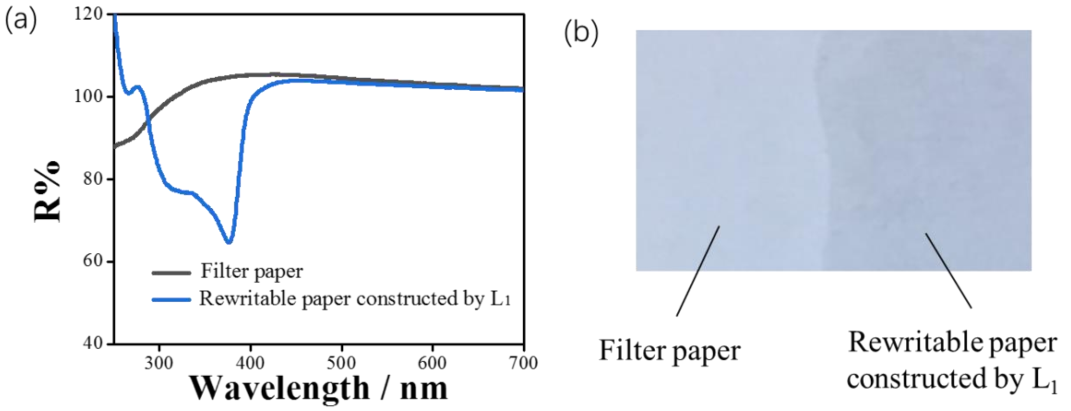

Supplementary Figure 16. (a) Reflective UV-vis spectra of filter paper and rewritable paper constructed with $\mathbf{L_1}$. (b) The photographs of filter paper and rewritable paper constructed by $\mathbf{L_1}$.

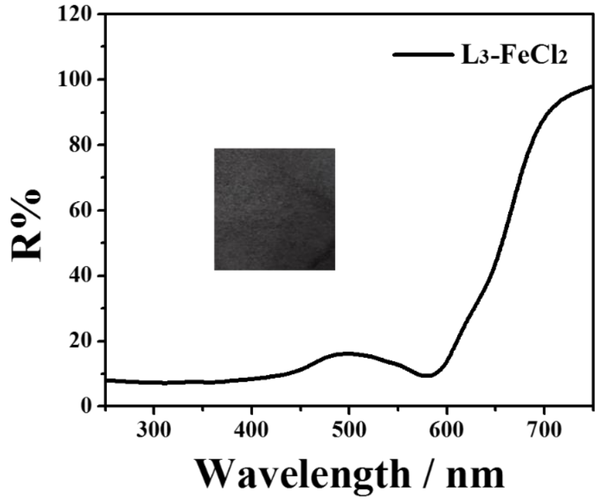

Supplementary Figure 17. The reflective UV-vis spectrum of rewritable paper constructed with **L_3** after addition of $FeCl_2$. Insert: The photograph of rewritable paper produced by addition of $FeCl_2$.

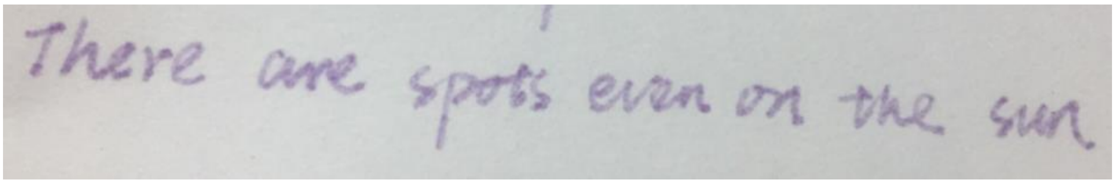

Supplementary Figure 18. Handwriting on rewritable paper by using $FeCl_2$ solution as ink.

2016.09

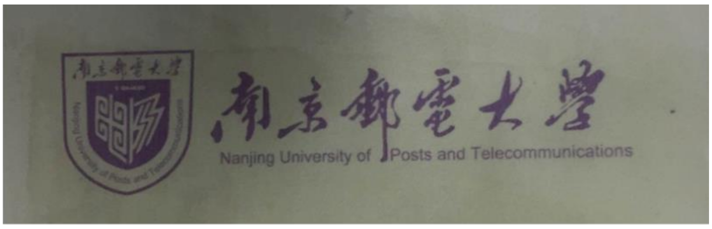

2017.06

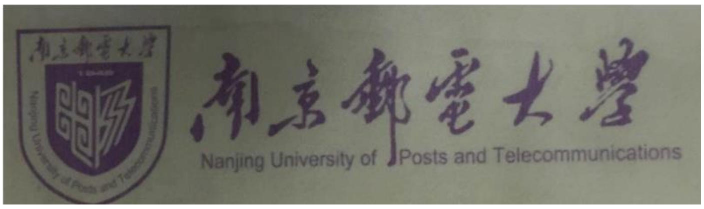

Supplementary Figure 19. Image of “Nanjing University of Posts and Telecommunications” printed using a customized black inkjet cartridge filled with $FeCl_2$ aqueous solution. The picture was taken in 2016.09 and 2017.06, respectively.

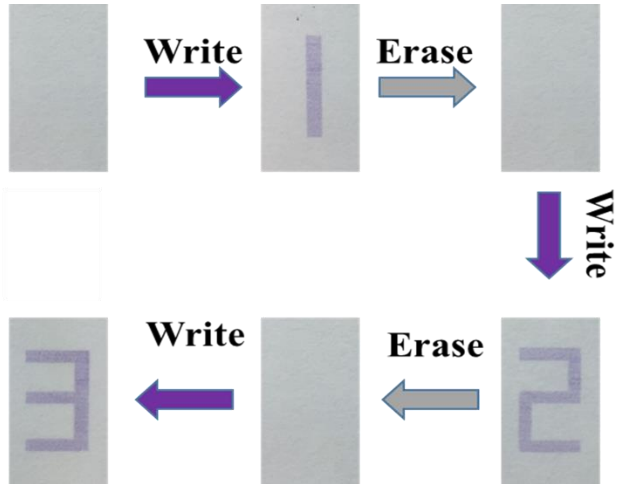

Supplementary Figure 20. Repeatedly recording Arabic numbers on the rewritable paper.

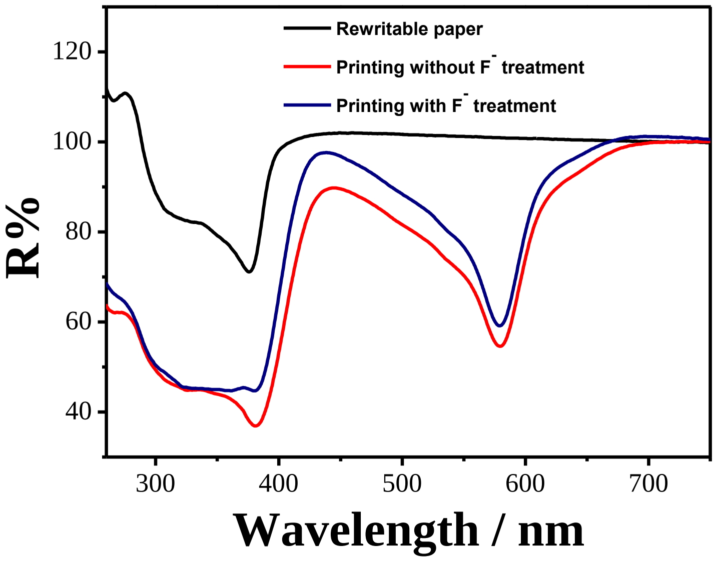

Supplementary Figure 21. Reflective UV-vis spectra of rewritable paper with and without pretreatment by F^- after printing.

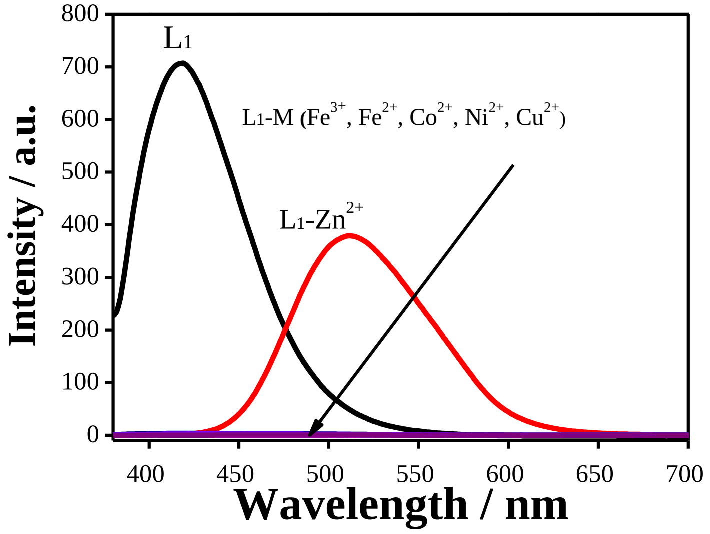

Supplementary Figure 22. The PL spectra of $\mathbf{L_1}$ by addition of different metal ions.

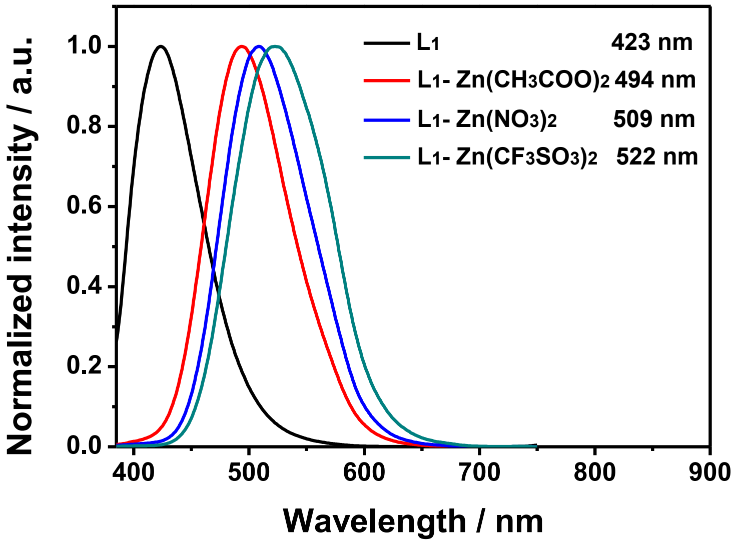

Supplementary Figure 23. Room-temperature normalized photoluminescence spectra of $\mathbf{L_1}$ and Zn(II) complexes in CH_2Cl_2 (10 μM).

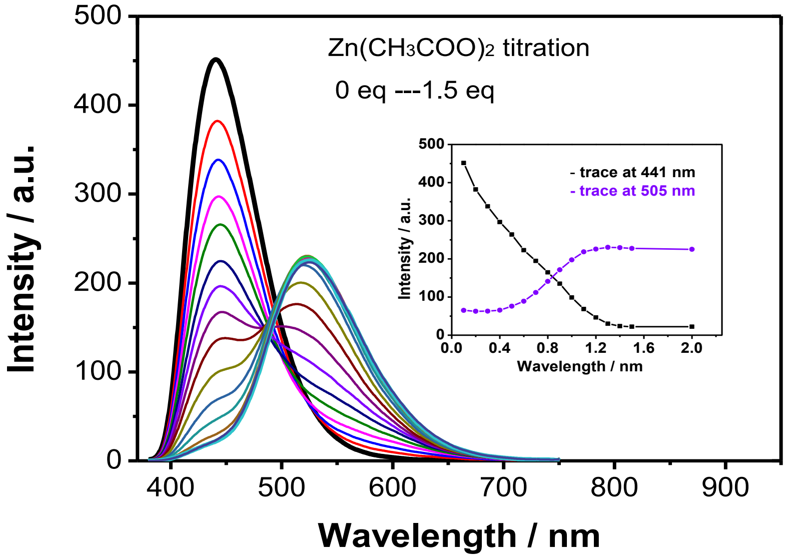

Supplementary Figure 24. Changes in the emission spectra of **L_2** upon $Zn(CH_3COO)_2$ titration (excitation: 365 nm). The inset indicates emission intensity traces at 441 nm and 505 nm.

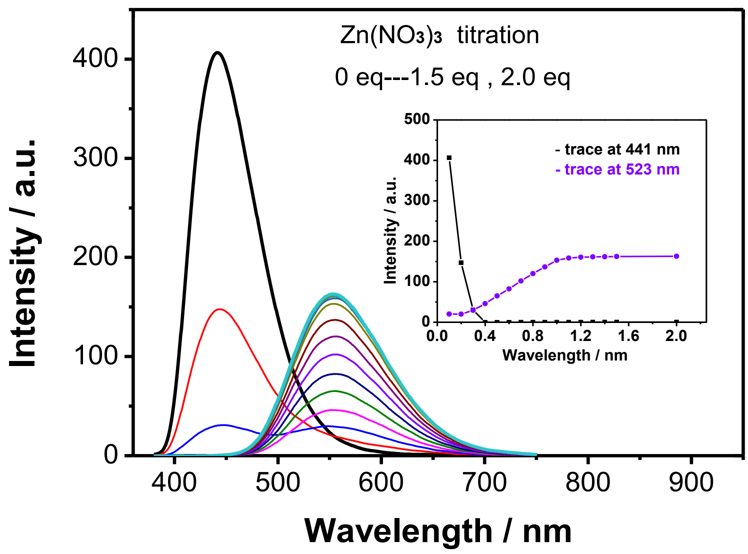

Supplementary Figure 25. Changes in the emission spectra of **L_2** upon $Zn(NO_3)_2$ titration (excitation: 365 nm). The inset indicates emission intensity traces at 441 nm and 523 nm.

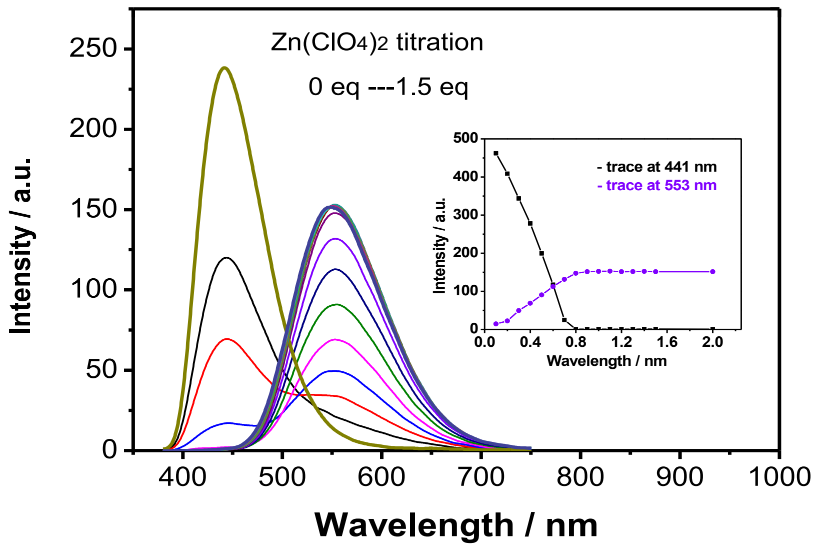

Supplementary Figure 26. Changes in the emission spectra of $\mathbf{L_2}$ upon $Zn(ClO_4)_2$ titration (excitation: 365 nm). The inset indicates emission intensity traces at 441 nm and 553 nm.

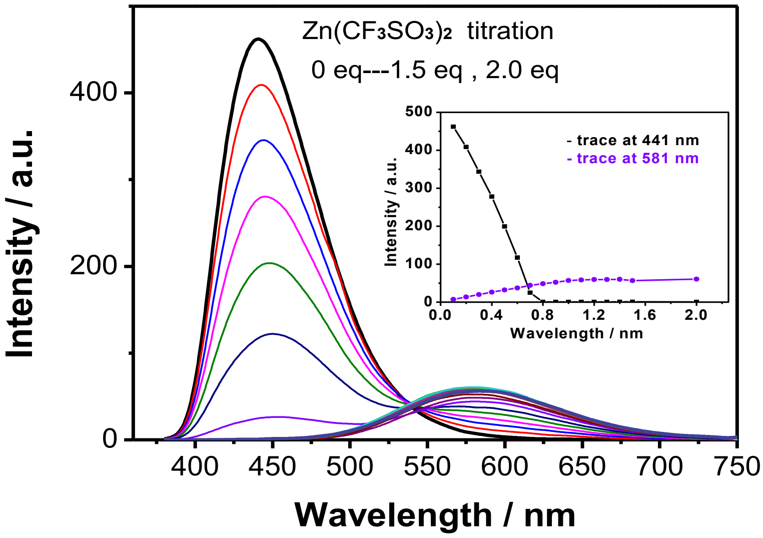

Supplementary Figure 27. Changes in the emission spectra of **L_2** upon $Zn(CF_3SO_3)_2$ titration (excitation: 365 nm). The inset indicates emission intensity traces at 441 nm and 581 nm.

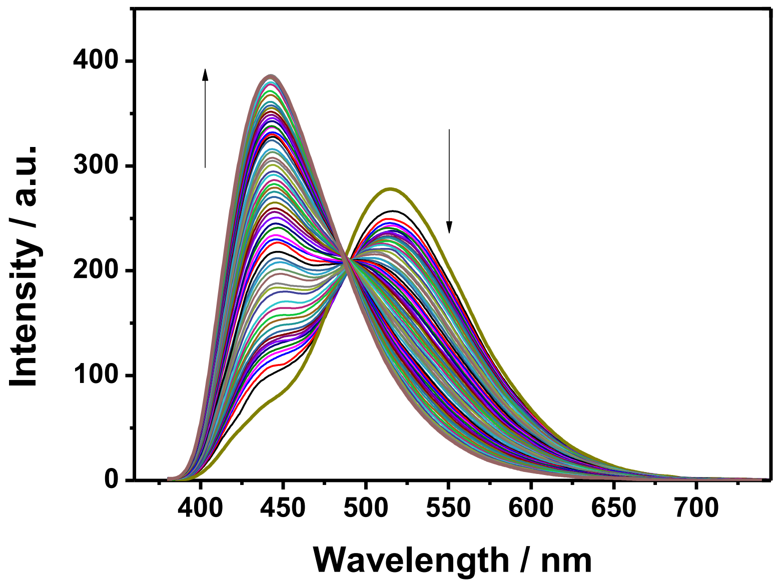

Supplementary Figure 28. Changes in the emission spectra of L_2–$Zn(CH_3COO)_2$ upon F^- titration (excitation: 365 nm). The inset indicates emission intensity traces at 505 nm and 441 nm.

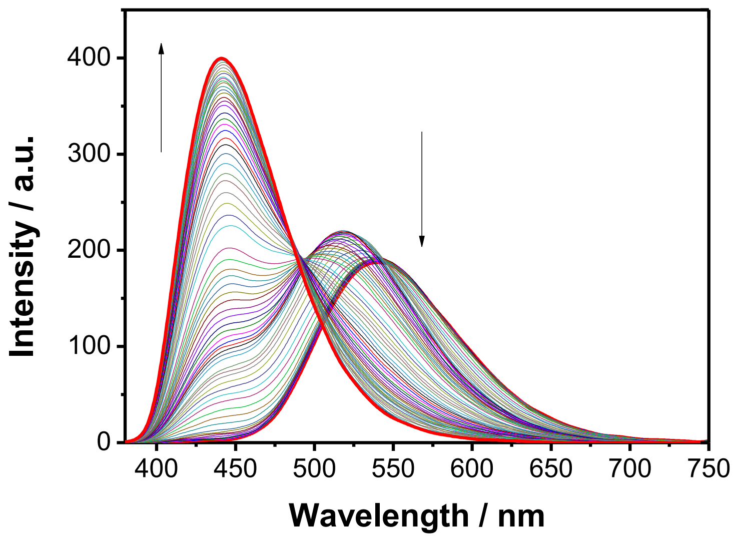

Supplementary Figure 29. Changes in the emission spectra of L_2-$Zn(NO_3)_2$ upon F^- titration (excitation: 365 nm). The inset indicates emission intensity traces at 441 nm and 523 nm.

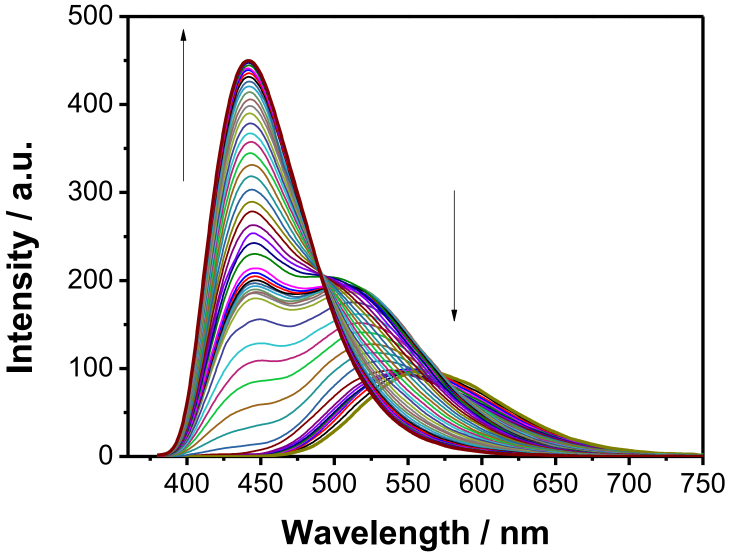

Supplementary Figure 30. Changes in the emission spectra of L_2-$Zn(ClO_4)_2$ upon F^- titration (excitation: 365 nm). The inset indicates emission intensity traces at 553 nm and 441 nm.

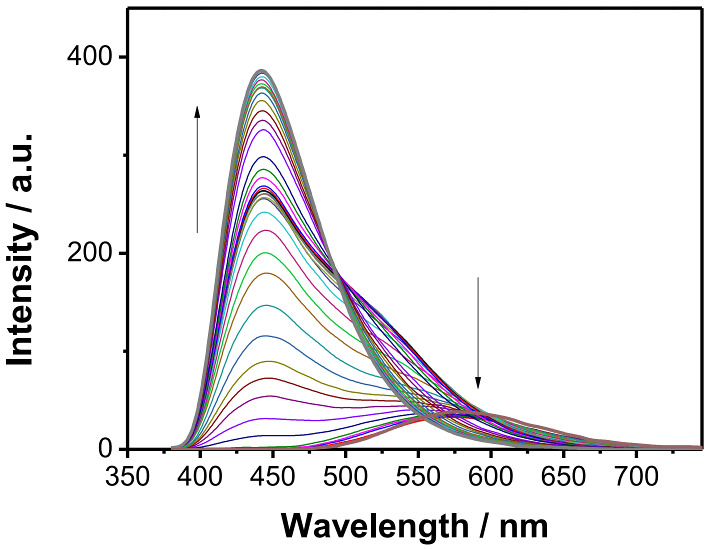

Supplementary Figure 31. Changes in the emission spectra of L_2-$Zn(CF_3SO_3)_2$ upon F^- titration (excitation: 365 nm). The inset indicates emission intensity traces at 581 nm and 441 nm.

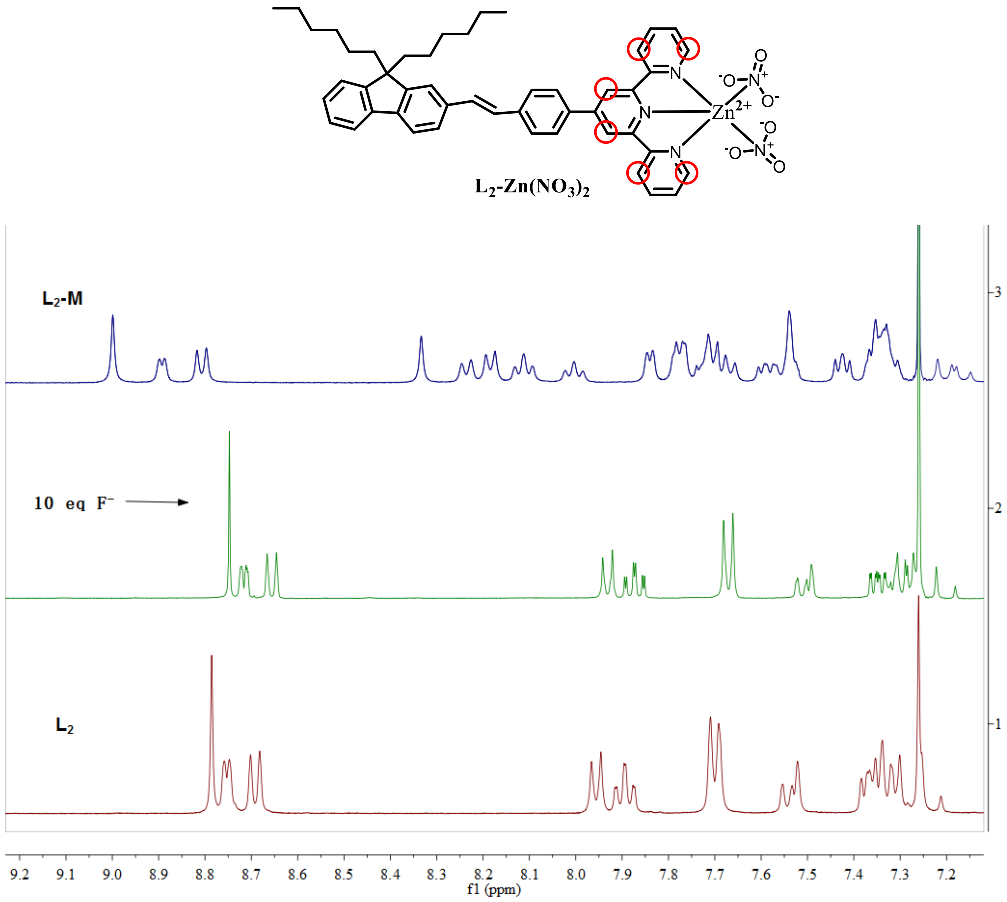

Supplementary Figure 32. Chemical structure of L_2-$Zn(NO_3)_2$ and ^1H NMR **L_2-$Zn(NO_3)_2$** with F^- titration in $CDCl_3$.

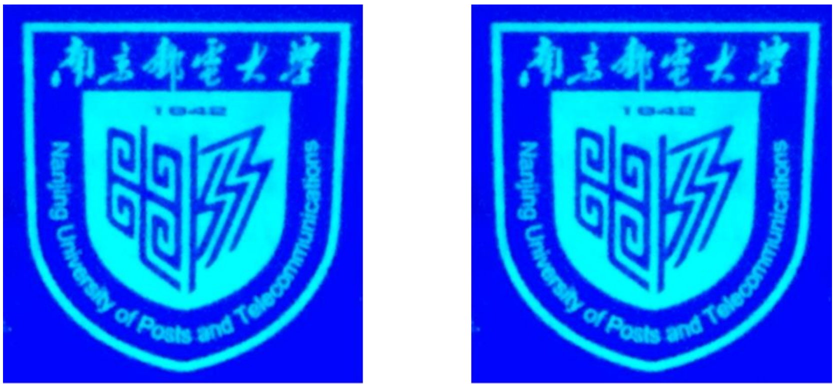

2016.01 2017.06

Supplementary Figure 33. Photo of the badge of “Nanjing University of Posts and Telecommunications” under UV light printed using a customized black inkjet cartridge filled with $Zn(CH_3COO)_2$ aqueous solution. The picture was taken in 2016.01 and 2017.06, respectively.

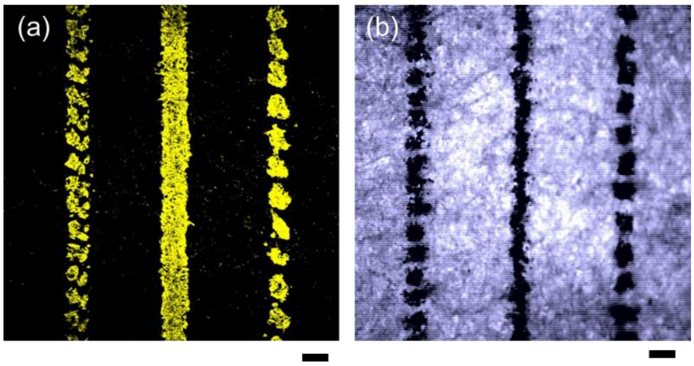

Supplementary Figure 34. The microscale patterns (thin line and dots line) were printed (a) on rewritable paper using $Zn(NO_3)_2$ as ink and (b) on A4 paper using commercial ink. Scale bar = 200 μm.

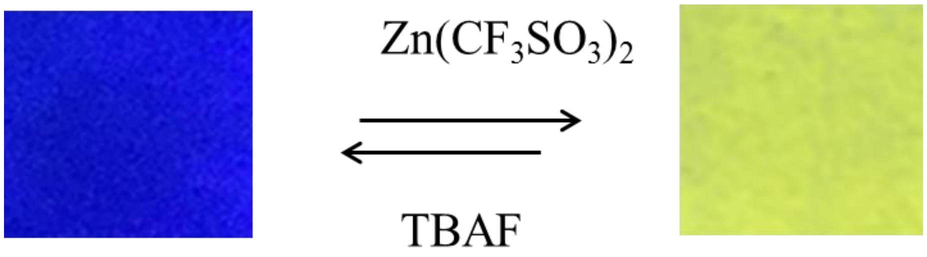

Supplementary Figure 35. Photographs of the yellow color printed by $Zn(CF_3SO_3)_2$ aqueous solution on rewritable paper followed erasing by immersing the rewritable paper into CH_2Cl_2 solution of TBAF.

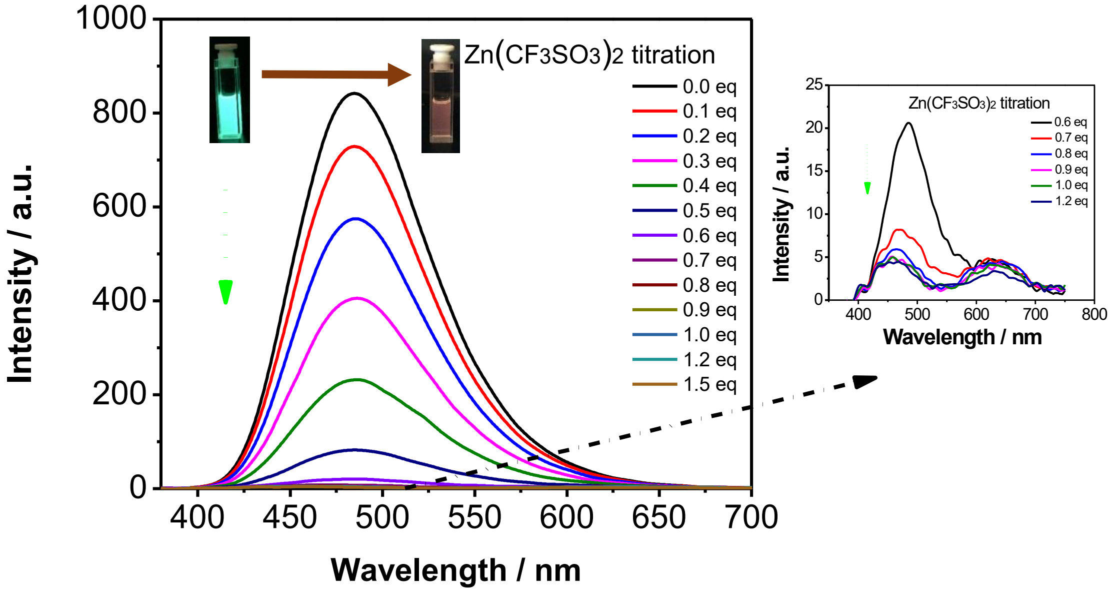

Supplementary Figure 36. Emission spectral change of $\mathbf{L_3}$ by addition of $Zn(CF_3SO_3)_2$ in THF solution.

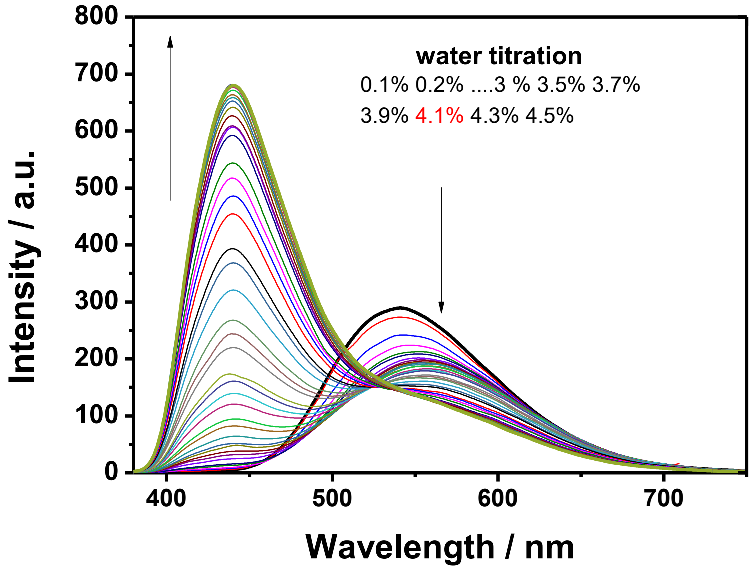

Supplementary Figure 37. PL spectra of complex **L_2-Zn** in THF-water mixtures with different water fractions.

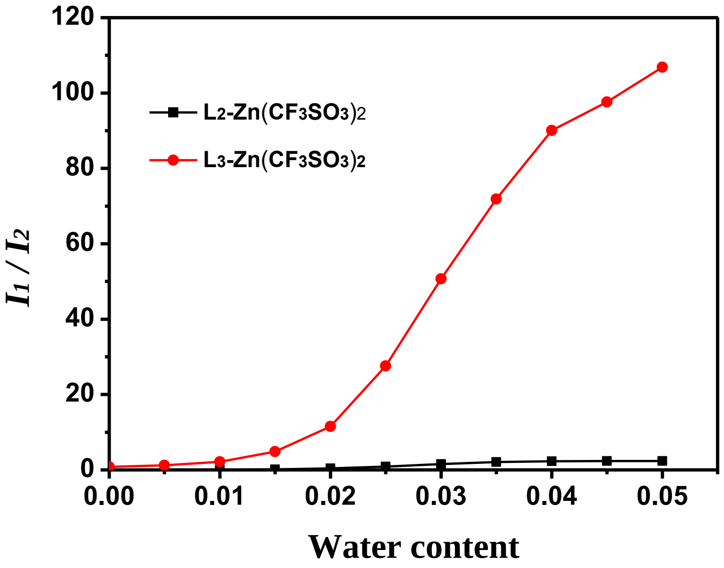

Supplementary Figure 38. The relative ratio of luminescence intensities of **L_2-Zn** and **L_3-Zn** over the water content range of 0–5 %.

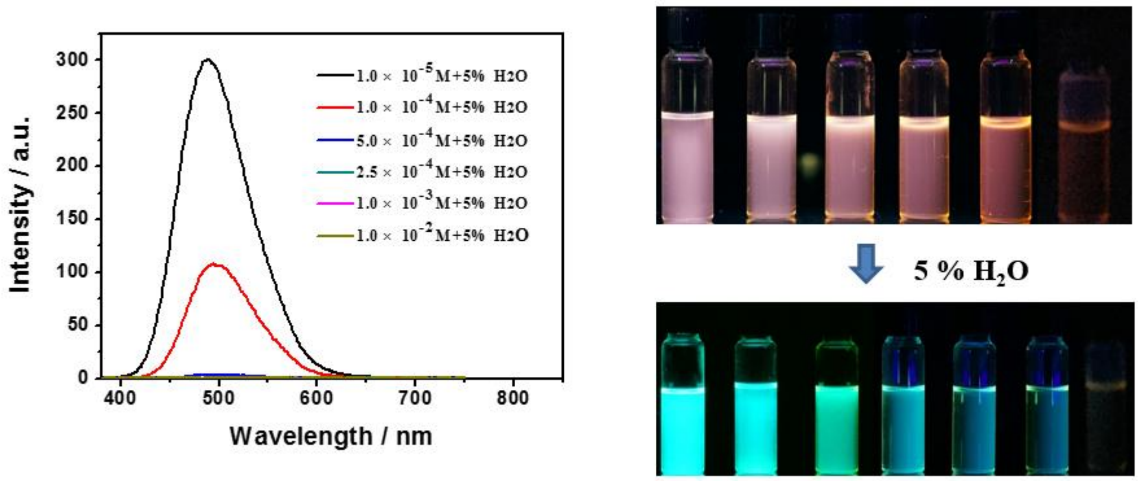

Supplementary Figure 39. (a) Emission spectra of **L3-Zn** in THF at different concentrations. (b) Photos of the emission colors of **L3-Zn** in THF at different concentrations.

2016.11

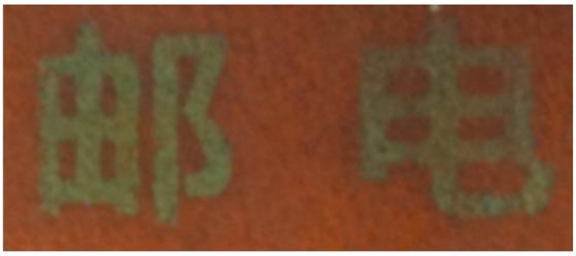

2017.06

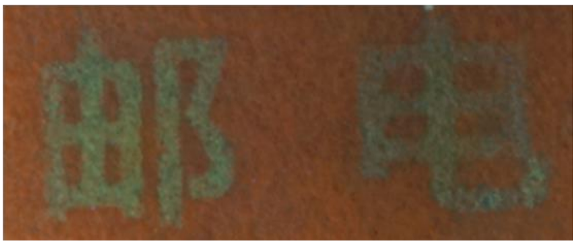

Supplementary Figure 40. Photo of Chinese characters under UV light printed using a customized black inkjet cartridge filled with pure aqueous solution. The picture was taken in 2016.11 and 2017.06, respectively.

Supplementary Table 1 Cost comparison between multicolour rewritable printing and HP ink-jet printing.

Project	HP ink-jet printing	Rewritable printing	Additional information
Printing Paper	A4 paper	Filter paper	Paper size 29.7×21 cm
Price (RMB) /sheet	0.04	0.08	
Ink cartridge	Ink	Metal salts solution	HP 1110 as an example
Price / sheet	0.1	0.005 (average)	
Other materials	-	$\mathbf{L_1}$ or $\mathbf{L_2}$ and PEG-PPG-PEG	
Dosage / sheet	-	$\mathbf{L_1}$ or $\mathbf{L_2}$ (9 mg) and PEG-PPG-PEG (285 mg)	Price of $\mathbf{L_1}$ or $\mathbf{L_2}$ is around 10 (RMB) / g
Price (RMB)	-	0.1	
Total costs for 8 sheets printing (RMB)	1.12	0.22	One sheet of rewritable paper can at least be used for 8 times

Supplementary Table 2 Cost comparison between water-jet rewritable printing and HP ink-jet printing.

Project	HP ink-jet printing	Rewritable printing	Additional information
Printing Paper	A4 paper	Filter paper	Paper size 29.7×21 cm
Price (RMB) /sheet	0.04	0.08	
Ink cartridge	Ink	H_2O	HP 1110 as an example
Price / sheet	0.1	-	
Other materials	-	**L_3-Zn** and PEG	
Dosage / sheet	-	**L_3-Zn** (1.5 mg) and PEG (285 mg)	Price of **L_3-Zn** is around 15 (RMB)/g
Price (RMB)	-	0.03	
Total costs for 8 sheets printing (RMB)	1.12	0.11	One sheet of rewritable paper can at least be used for 8 times

RMB is the abbreviation for Renminbi Yuan.

One cartridge can be limited to print 480 sheets for black mark and 200 sheets for colour mark, calculated by 5 % cover printing for each sheet.

Supplementary Table 3 Cell viabilities of HeLa cells. Cell viability values assessed using an MTT test versus incubation concentrations.

	1 μM	5 μM	10 μM	50 μM
**L_1**	1.059 ± 0.052	0.998 ± 0.091	0.989 ± 0.072	0.807 ± 0.062
**L_2**	0.993 ± 0.066	0.982 ± 0.048	0.878 ± 0.093	0.760 ± 0.059
**L_3**	0.986 ± 0.035	0.946 ± 0.020	0.831 ± 0.136	0.836 ± 0.128
**L_1-$Fe(NO_3)_3$**	0.961 ± 0.030	0.916 ± 0.091	0.886 ± 0.058	0.746 ± 0.068
**L_1-$Co(NO_3)_2$**	1.000 ±0.057	0.923 ± 0.076	0.923 ± 0.040	0.964 ± 0.094
**L_1-$Ni(NO_3)_2$**	0.969 ± 0.102	0.917 ± 0.047	0.814 ± 0.025	0.796 ± 0.041
**L_1-$FeCl_3$**	0.952 ± 0.095	0.917 ± 0.108	0.958 ± 0.069	0.842 ± 0.007
**L_1- $FeCl_2$**	0.961 ± 0.064	0.836 ± 0.049	0.918 ± 0.088	0.789 ± 0.021
**L_1-$Zn(NO_3)_2$**	1.178 ± 0.079	1.078± 0.150	1.034± 0.059	0.757± 0.060
**L_1-$CuCl_2$**	0.986 ± 0.036	0.962 ± 0.075	0.891 ± 0.010	0. 762 ± 0.017
**L_2-$Zn(ACO)_2$**	0.852 ± 0.041	0.837 ± 0.052	0.965 ± 0.084	0.880 ± 0.084
**L_2-$Zn(NO_3)_2$**	0.978 ± 0.131	0.959 ± 0.041	0.850 ± 0.042	0.775 ± 0.069
**L_2-$Zn(ClO_4)_2$**	1.042 ± 0.046	1.162 ± 0.084	1.103 ± 0.051	0.867 ± 0.039
**L_2-$Zn(CF_3SO_3)_2$**	1.010 ± 0.040	1.054 ± 0.032	0.973 ± 0.064	0.828 ± 0.022
**L_3-$Zn(CF_3SO_3)_2$**	0.988 ± 0.042	0.959 ± 0.044	0.875 ± 0.022	0.763 ± 0.030

Supplementary Methods

Synthetic Procedures

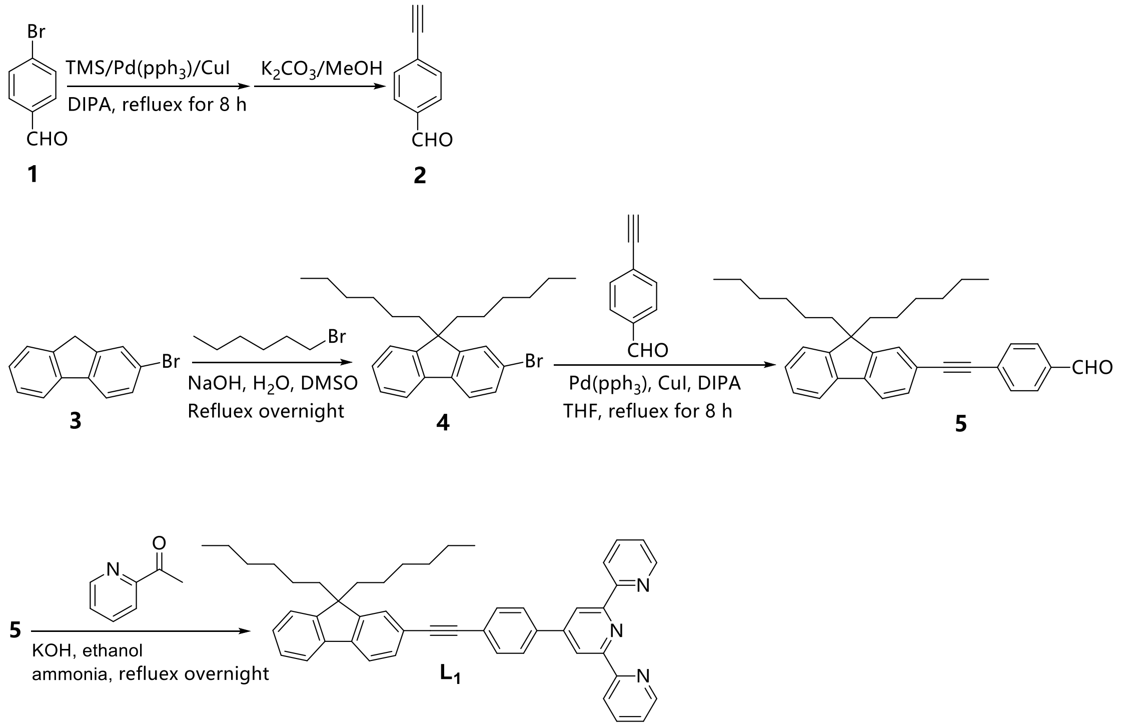

Synthesis of L_1

Synthesis of **5**: Compounds **2** and **4** were synthesized according to the previous method.[1,2] The mixture of **2** (0.6 mmol, 193 mg), **4** (0.6mmol, 77 mg), $Pd(PPh_3)_4$ (0.018 mmol, 2.1mg), CuI (0.03 mmol, 0.6 mg), THF (15 mL) and N-(1-Methylethyl)-2-propanamine (DIPA) (30 mL) was refluxed under nitrogen atmosphere at 80 ºC for 8 h. After cooling to room temperature, the solution was extracted with water and dichloromethane. The organic phase was concentrated and then purified by silica gel chromatograph using petroleum ether and dichloromethane (10 : 1, v : v) to give the light yellow soild. Yield: 52 %. ^{1}H NMR (400 MHz, $CDCl_3$): δ 10.03 (s, 1H), 7.90-7.87 (d, J = 8 Hz, 2H), 7.73-7.69 (m, 4H), 7.56-7.54 (m, 2H), 7.38-7.32 (m, 3H), 2.01-1.97 (t, J = 16.8 Hz, 4H), 1.16-0.98 (m, 12H), 0.79-0.75 (d, J = 14.4 Hz, 6H), 0.68-0.54 (m, 4H); ^{13}C NMR (100 MHz, $CDCl_3$): δ 191.55, 151.20, 151.01, 142.31, 140.32, 135.38, 132.14, 130.99, 129.98, 129.75, 127.90, 127.07,

126.23, 123.04, 120.65, 120.26, 119.87, 94.95, 88.72, 55.29, 40.49, 31.63, 29.82, 23.83, 22.71, 14.13; MS (MALDI-TOF) (m/z): calcd. for $C_{34}H_{38}O$, 462.68; found: 462.84.

Synthesis of **L_1**: The mixture of **5** (0.5 mmol, 203 mg), 1-(Pyridin-2-yl)ethanone (1.8 mmol, 0.2mL), KOH (1.8 mmol, 0.1 mg), ethyl alcohol (15 mL) and ammonium hydroxide (5 mL) was refluxed at 55 °C for 8 h. The mixture was cooled to room temperature and diluted with water (300 mL), and then extracted with dichloromethane. The product was isolated as white crystals by the recrystallization from ethanol/dichloromethane. ^{1}H NMR (400 MHz, $CDCl_3$): δ 8.77 (s, 2H), 8.75-8.74 (d, J = 4 Hz, 2H), 8.70-8.68 (d, J = 7.6 Hz, 2H), 7.95-7.87 (m, 4H), 7.73-7.69 (m, 4H), 7.57-7.55 (d, J = 9.2 Hz, 2H), 7.39-7.30 (m, 5H), 2.01-1.97(t, J = 16.8 Hz, 4H), 1.16-0.99 (m, 12H), 0.79-0.75 (d, J = 14.4 Hz, 6H), 0.69-0.56 (m, 4H); ^{13}C NMR (100 MHz, $CDCl_3$): δ 156.29, 156.19, 151.21, 150.98, 149.56, 149.30, 141.77, 140.53, 138.13, 137.05, 132.25, 130.82, 127.70, 127.38, 126.92, 126.16, 124.33, 124.05, 122.98, 121.52, 121.34, 120.17, 119.81, 118.73, 92.19, 89.24, 55.26, 40.51, 31.67, 29.87, 23.87, 22.75, 14.15; MS (MALDI-TOF) (m/z): calcd. for $C_{48}H_{47}N_3$, 665.93; found: 665.54.

Synthesis of L_2

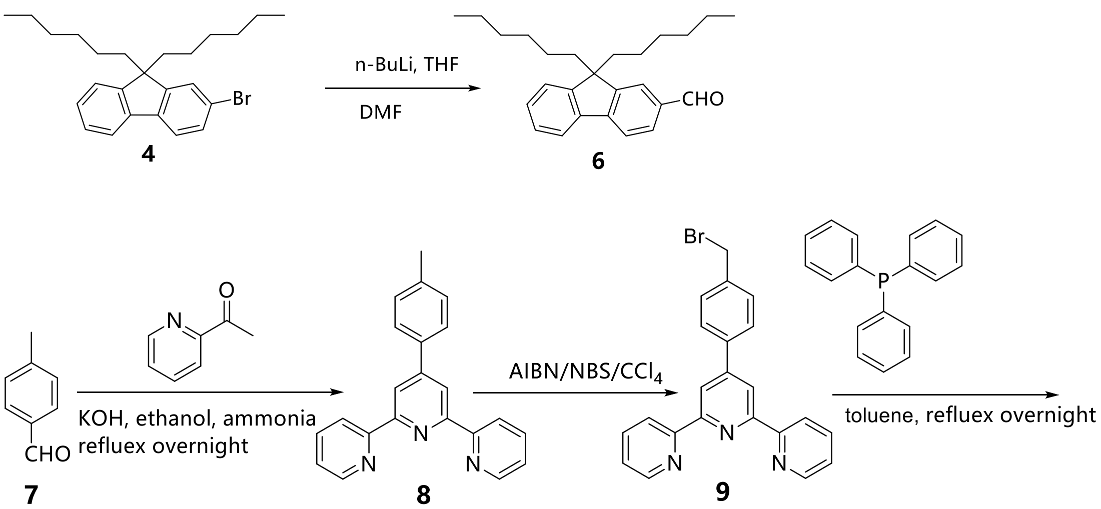

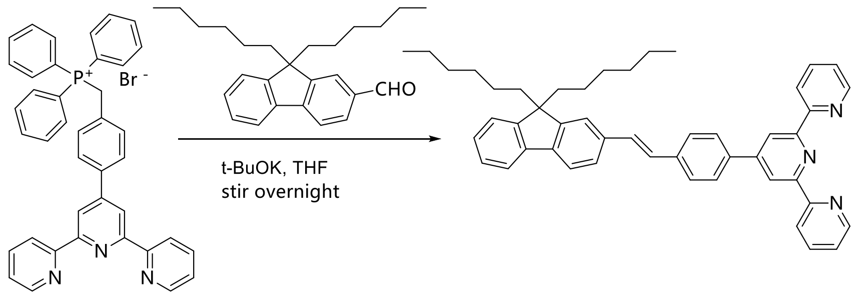

10 **L_2**

Synthesis of **6**: The bromide at the 2-position was activated by adding one equivalent of *n*-BuLi in dry THF at -78 ºC and excess of *N*,*N*-dimethylformamide (DMF) and stirring the reaction mixture for overnight at room temperature. Subsequently, the solution was quenched with water and washed with dichloromethane. The organic phase was concentrated and then purified by silica gel chromatograph using petroleum ether and dichloromethane (100 : 1, v/v) to give the yellow transparent liquid. Yield: 60 %. ^{1}H NMR (400 MHz, DMSO-d_6): δ 10.04 (s, 1H), 8.05-8.34(d, J = 7.6H, 1H), 7.96-7.9 (m, 3H), 7.51-7.37 (m, 3H), 2.12-1.95 (m, 4H), 1.06-0.90 (m, 12H), 0.71-0.67 (t, J = 14Hz), 0.48-0.41 (m, 4H).

Synthesis of **10**: The mixture of **9**[3] (2.4 mmol, 1 g), triphenylphosphine (6 mmol, 1.55 g) and toluene (40 mL) was refluxed under nitrogen atmosphere (100 mL) at 90 ºC for 10 h. After cooling to room temperature, the resulting white precipitate was collected by filtration and washed with toluene. Yield: 88%.^{1}H NMR (400 MHz, DMSO-d_6): δ 8.75-8.74 (d, J = 4.8 Hz, 2H), 8.68-8.66 (m, 4H), 8.06-8.02 (t, J = 15.2 Hz, 2H), 7.95-7.91 (t, J = 14.4 Hz, 3H), 7.85-7.70 (m, 13H), 7.55-7.52 (m, 2H), 7.26-7.12 (m, 2H), 5.31-5.26 (m, 2H).

Synthesis of **L_2**: The mixture of compound **10** (1.8 mmol, 1.2 g), compound **6** (1.8 mmol, 0.58 g), *t*-BuOK(3.6 mmol, 0.4 g) and THF (40 mL) was stirred under nitrogen atmosphere at 25 ºC for 10 h. Then the solution was extracted with water and

dichloromethane, and evaporated to yield a gray sludge. The product was isolated as white crystals by recrystallization from ethanol. Yield: 33 %. ^{1}H NMR (400 MHz, $CDCl_3$) δ: 8.786 (s, 2H), 8.76-8.75 (d, *J* = 4.8 Hz, 2H), 8.70-8.68 (d, *J* = 8 Hz, 2H), 7.97-7.95 (d, *J* = 8.4 Hz, 2H), 7.92-7.87 (m, 2H), 7.71-7.69 (d, *J* = 6.8 Hz, 4H), 7.55-7.22 (m, 2H), 7.38-7.21(m, 7H), 2.03-1.98 (t, *J* = 16.4 Hz, 4H)，1.14-1.05 (m, 12H), 0.78-0.75 (t, *J* = 14 Hz, 6H), 0.72-0.57 (m, 4H); ^{13}C NMR (100 MHz, $CDCl_3$) δ: 151.43, 151.17, 149.84, 149.28, 141.34, 140.91, 138.54, 137.32, 137.03, 136.20, 130.38, 127.76, 127.28, 127.24, 127.09, 126.93, 125.87, 123.98, 123.00, 121.53, 121.01, 120.06, 119.86, 118.64, 55.17, 40.64, 31.66, 29.89, 23.89, 22.75, 14.16; MS (MALDI-TOF) (m/z): calcd. for $C_{48}H_{49}N_3$, 667.94; found: 666.52.

Synthesis of L$_3$

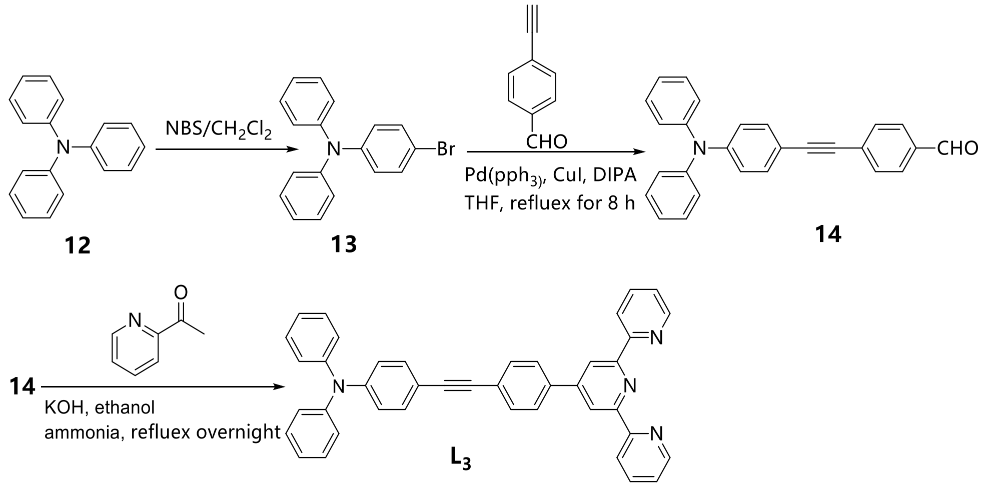

Synthesis of **13**: The mixture of compound **12** (5.3 mmol, 1.3 g), n-bromosuccinimide (4.4 mmol, 757 mg) was refluxed under nitrogen atmosphere at 80 ºC for 8 h. ^{1}H NMR (400 MHz, $CDCl_3$): δ 7.35-7.20 (m, 6H), 7.11-6.99 (m, 6H), 6.97-6.90 (m, 2H).

Synthesis of **14**: The procedure was similar to that for the preparation of compound **5**, except that compound **13** (0.6 mmol, 194 mg) was used instead. Subsequent the organic phase was concentrated and then purified by silica gel chromatograph using petroleum ether and dichloromethane (5 : 1, v/v) to give the light green solid. Yield:

57.3 % . ^{1}H NMR (400 MHz, DMSO-d_6): δ 10.01 (s, 1H), 7.93-7.91 (d, *J* = 8.4 Hz, 2H), 7.72-7.70 (d, *J*=8.4 Hz, 2H), 7.46-7.44 (d, *J* = 8.8 Hz, 2H), 7.38-7.34 (t, *J* = 15.6 Hz, 4H), 7.16-7.09 (m, *J* = 8.8, 6H), 6.91-7.89 (d, *J* = 8.8 Hz, 2H).

Synthesis of $\mathbf{L_3}$: The procedure was similar to that for the preparation of $\mathbf{L_1}$, except that compound **14** (0.5 mmol, 187 mg) was used instead. The product was isolated as yellow crystals by recrystallization from ethanol/dichloromethane. ^{1}H NMR (400 MHz, THF-d_8**)** δ: 8.78-8.75 (t, *J* = 11.6 Hz, 4H), 8.70-8.68 (d, *J* = 8 Hz, 2H), 8.08-7.99 (m, 4H), 7.73-7.71 (d, *J* = 8 Hz, 2H), 7.56-7.53 (t, *J* = 12.4 Hz, 2H), 7.49-7.46 (d, *J* = 8.4 Hz, 2H), 7.39-7.35(t, *J* = 16.4 Hz, 4H), 7.16-7.10 (m, 6H)， 6.93-6.91 (d, *J* = 8.4 Hz 2H); ^{13}C NMR (100 MHz, $CDCl_3$) δ: 156.24, 156.10, 149.48, 149.24, 148.17, 147.26, 137.81, 136.98, 132.73, 132.08, 129.53, 127.31, 125.15, 124.53, 123.99, 123.72, 122.31, 121.47, 118.70, 115.94, 91.44, 88.55**;** MS (MALDI-TOF) [m/z]: calcd. for $C_{41}H_{28}N4$, 576.70; found: 575.45.

^{1}H NMR spectrum of L$_1$

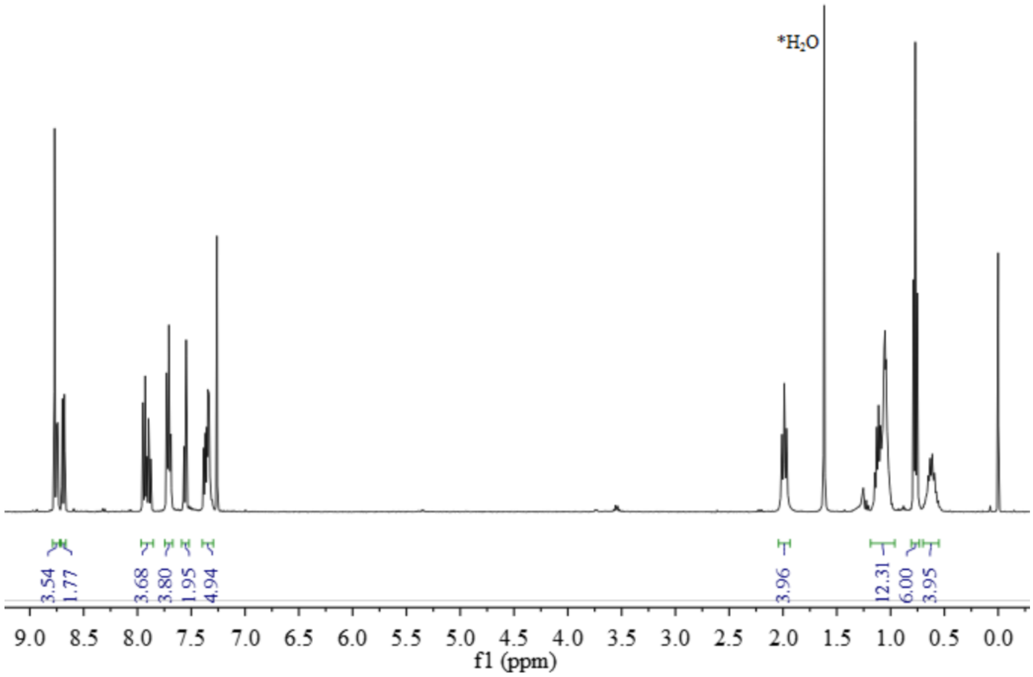

^{13}C NMR spectrum of L_1

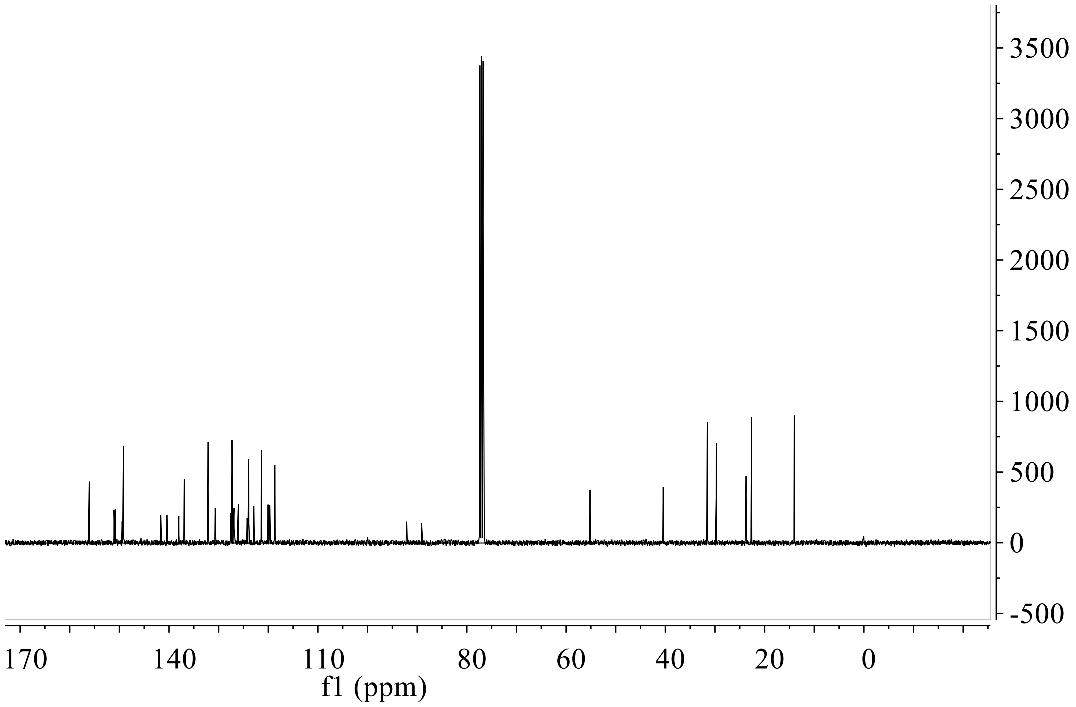

^{1}H NMR spectrum of L_2

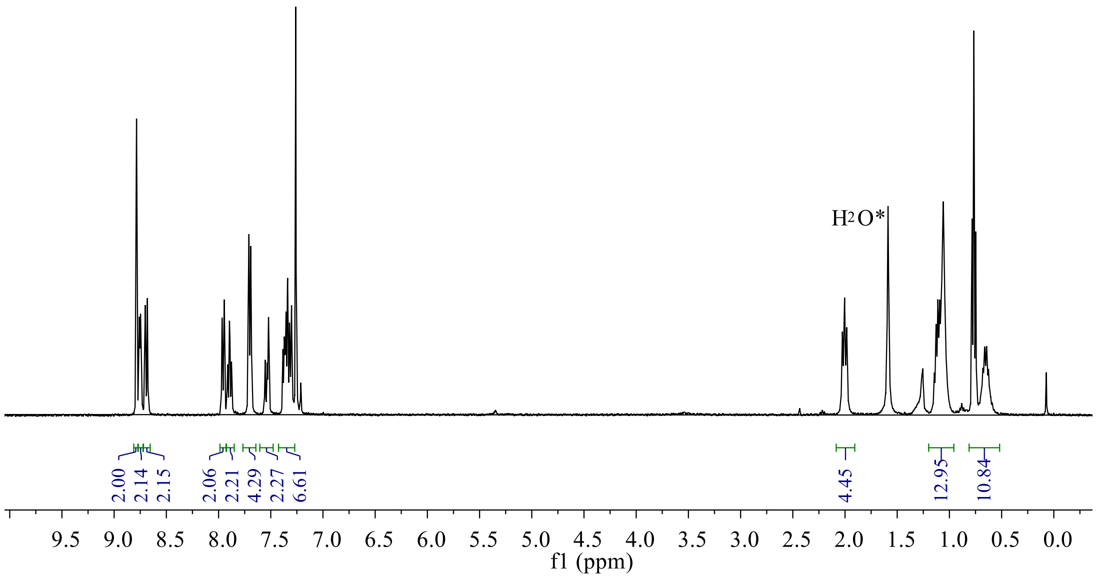

^{13}C NMR spectrum of L_2

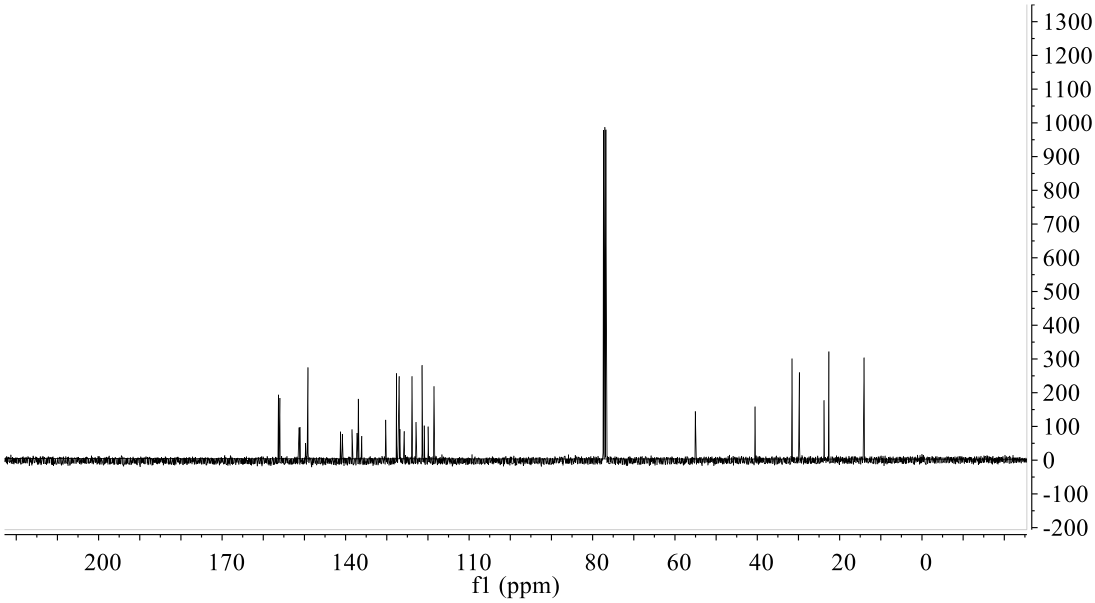

^{1}H NMR spectrum of L_3

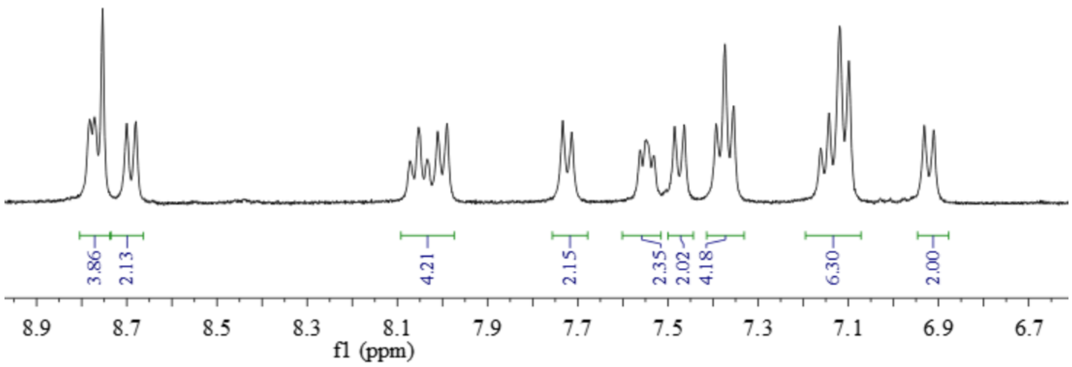

^{13}C NMR spectrum of L_3

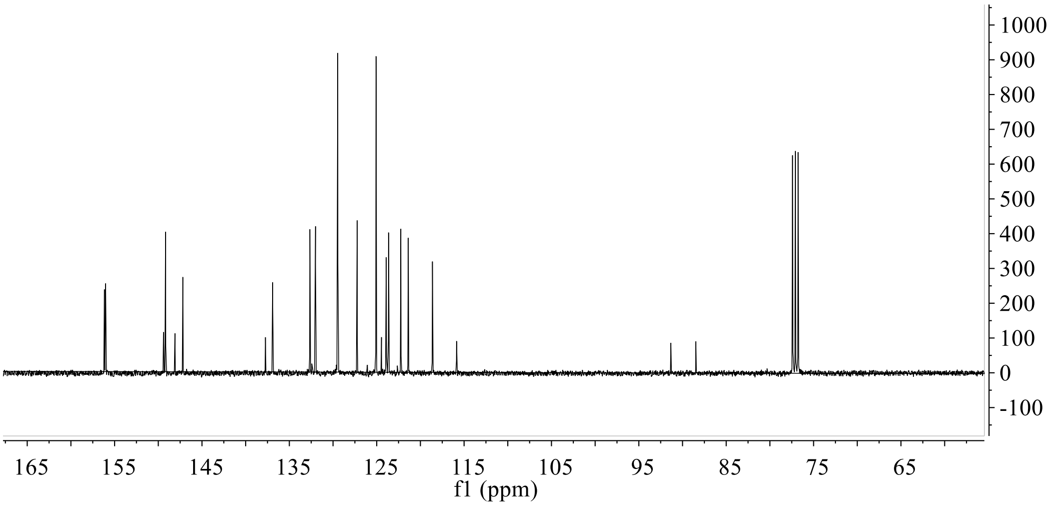

^{1}H NMR spectra of L_2 –$Zn(CH_3COO)_2$

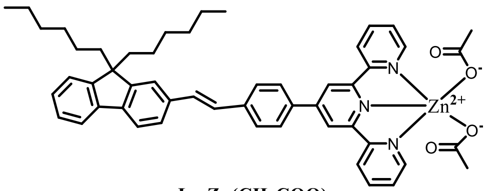

L_2-$Zn(CH_3COO)_2$

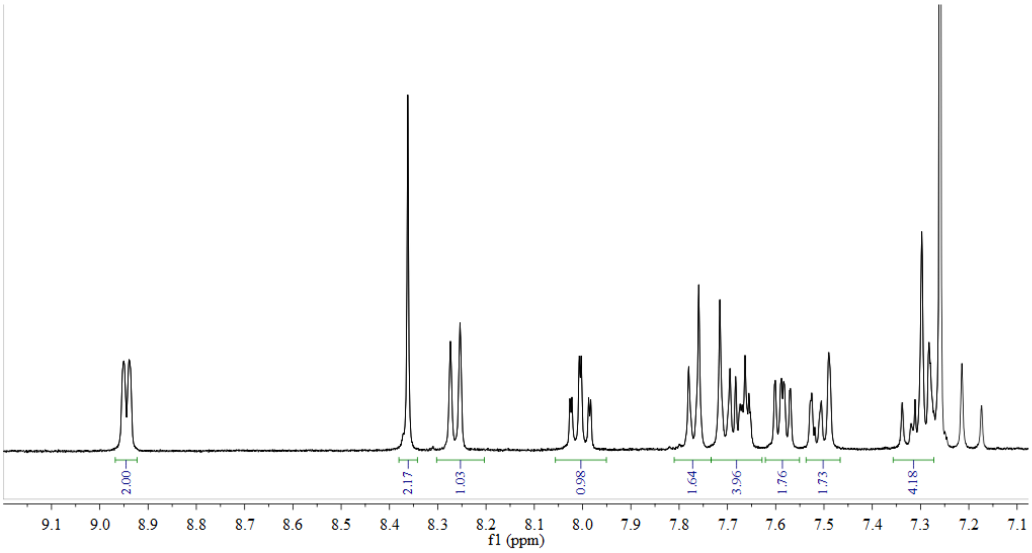

^{1}H NMR spectra of L_2 –$Zn(NO_3)_2$

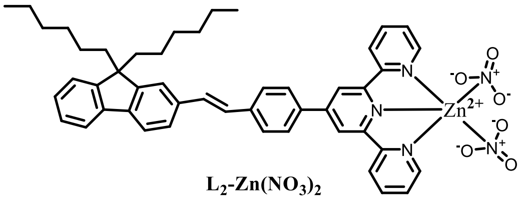

L_2-$Zn(NO_3)_2$

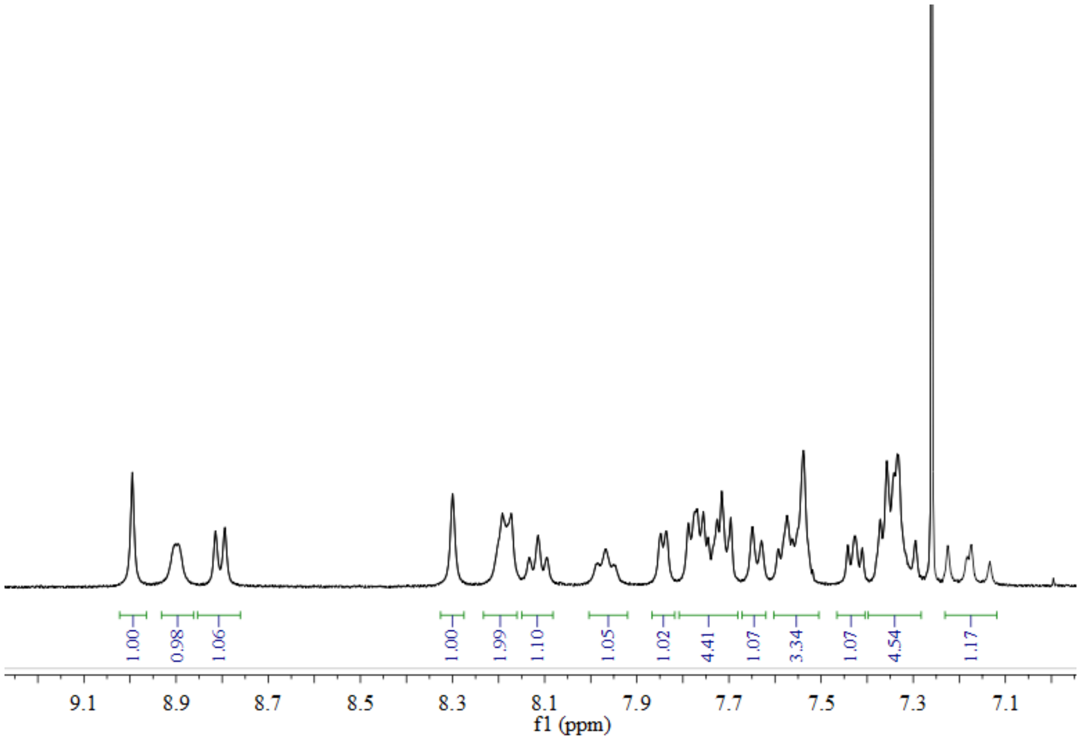

^{1}H NMR spectra of L_2 –$Zn(ClO_4)_2$

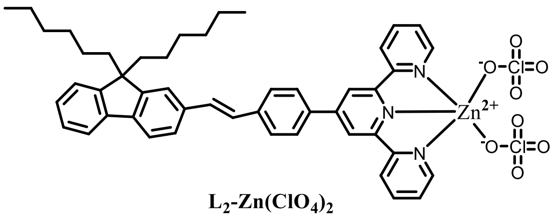

L_2-$Zn(ClO_4)_2$

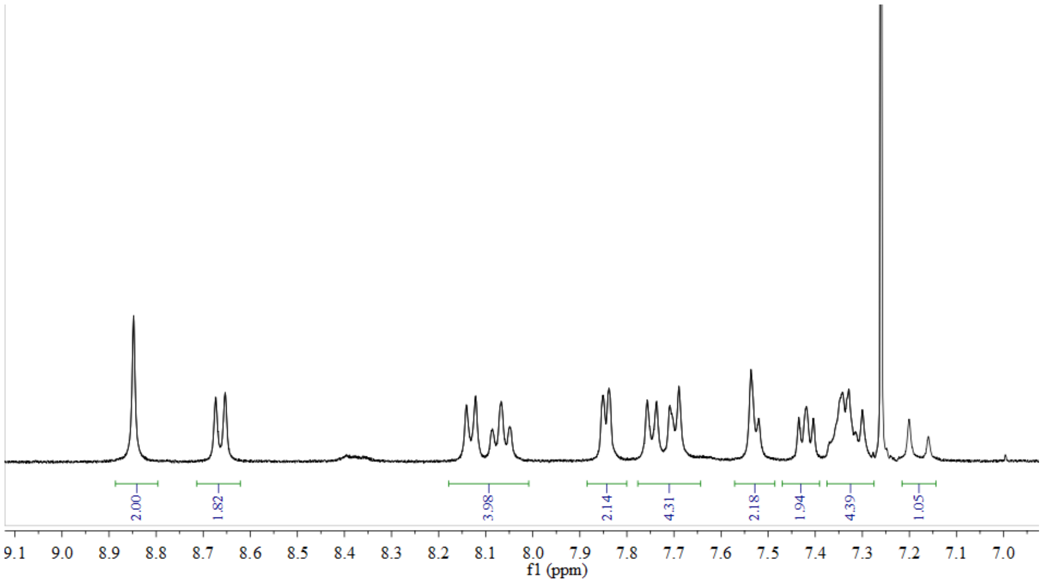

^{1}H NMR spectra of L_2–$Zn(CF_3SO_3)_2$

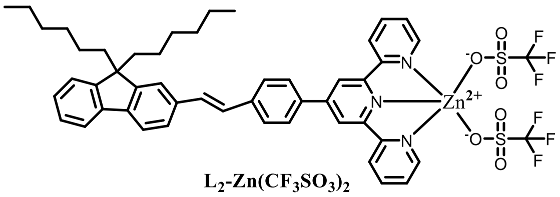

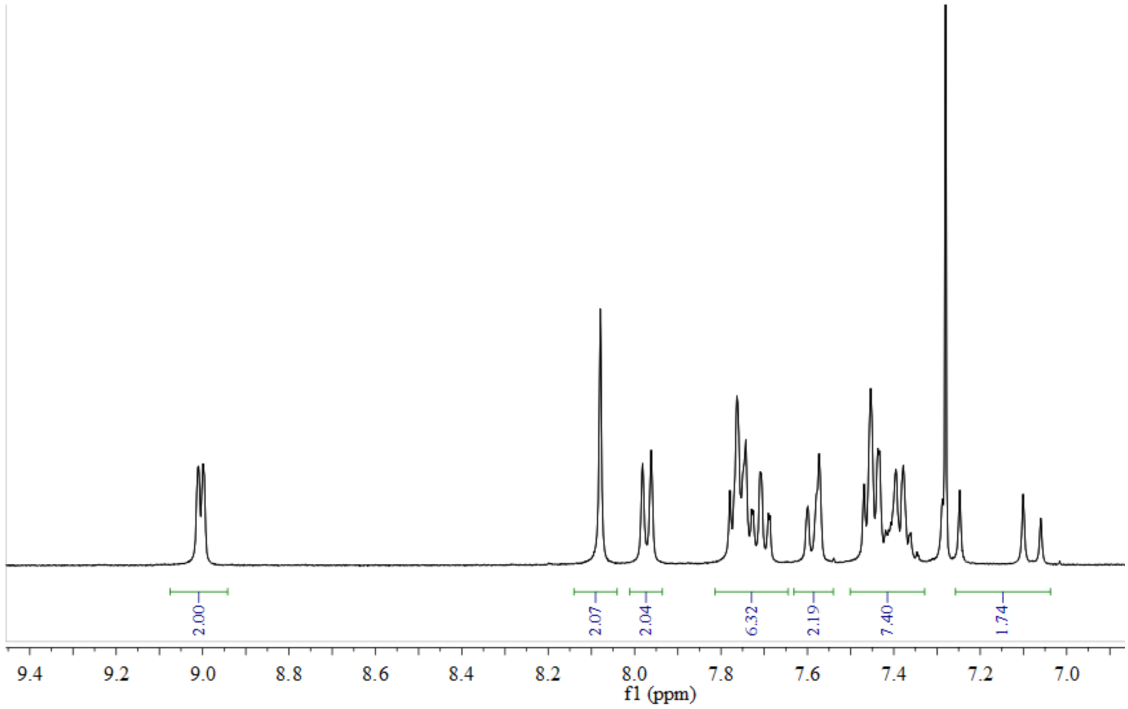

^{1}H NMR spectra of L_3 –$Zn(CF_3SO_3)_2$

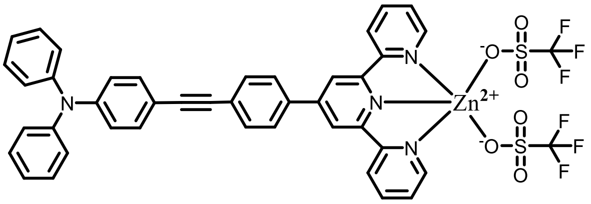

L_3-$Zn(CF_3SO_3)_2$

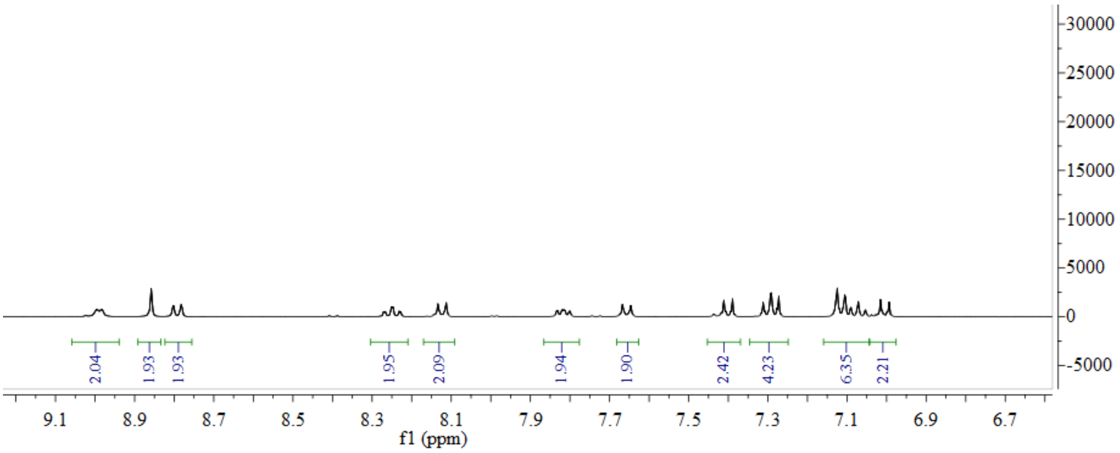

Supplementary References

[1] Yuan, S. C., Chen, H. B., Zhang, Y., & Pei, J. Rigid linear and star-shaped π -conjugated 2, 2’ :6’,2’’ -terpyridine ligands with blue emission. *Org. Lett.* **8**, 5701-5704 (2006).

[2] Byrne, J. P. *et. al.* Synthesis, structural, photophysical and electrochemical studies of various d-metal complexes of btp [2,6-bis(1,2,3-triazol-4-yl)pyridine] ligands that give rise to the formation of metallo-supramolecular gels. *Dalton Trans.* **43**, 196-209 (2014).

[3] Ohr, K., McLaughlin, R. L. & Williams, M. E. Redox behavior of phenyl-terpyridine-substituted artificial oligopeptides cross-linked by Co and Fe. *Inorg. Chem.* **3**, 965-974 (2007).
